# A taxonomic framework of music-based intervention components to guide research: recommendations from the NIH U24 music research networks

**DOI:** 10.3389/fpsyg.2026.1876240

**Published:** 2026-09-09

**Authors:** Sheri L. Robb, Elizabeth Harman, Debra S. Burns, Jeffery A. Dusek, Julene K. Johnson, Samuel N. Rodgers-Melnick, Kamal R. Chémali, Joseph J. Schlesinger, Mirian Ramirez, Lynn Gumert, Sarah Biedka, Jannatul Khawsar, Michael Kiley, Joanne Loewy, Joke Bradt

**Affiliations:** 1 School of Nursing, Indiana University, Indianapolis, IN, United States; 2 School of Medicine, Indiana University, Indianapolis, IN, United States; 3 College of Communication and Fine Arts, University of Memphis, Memphis, TN, United States; 4 Susan Samueli Integrative Health Institute, University of California, Irvine, Irvine, CA, United States; 5 Department of Medicine, University of California, Irvine, Irvine, CA, United States; 6 Division of Geriatrics, School of Medicine, University of California, San Francisco, San Francisco, CA, United States; 7 University Hospitals of Cleveland, Cleveland, OH, United States; 8 School of Medicine, Case Western Reserve University, Cleveland, OH, United States; 9 Vanderbilt University Medical Center, Nashville, TN, United States; 10 Ruth Lilly Medical Library, School of Medicine, Indiana University, Indianapolis, IN, United States; 11 Department of Creative Arts Therapies, Drexel University, Philadelphia, PA, United States; 12 Louis Armstrong Center for Music and Medicine, Icahn School of Medicine at Mount Sinai, New York, NY, United States

**Keywords:** music, music neuroscience, music perception, music-based intervention, taxonomy

## Abstract

**Background:**

As research on music and health expands across disciplines, inconsistent terminology limits communication, replication, and mechanistic discovery. The absence of a shared lexicon obscures comparisons across studies and hinders identification of the specific music-based intervention (MBI) components and mechanistic pathways responsible for change in health-related outcomes.

**Objective:**

To address this gap, a multidisciplinary working group representing four U.S. National Institutes of Health (NIH) U24 Music Research Networks developed and validated the Music-Based Intervention (MBI) Taxonomy.

**Methods:**

Using an iterative, expert consensus process, the working group developed an initial framework of MBI components, terms, and definitions. The draft taxonomy was subsequently validated, interrogated, and refined through a two-part systematic review of 200 studies, including 100 clinical MBI and 100 laboratory-based music studies, using a phased, iterative, consensus-based process.

**Results:**

The resulting taxonomy provides a hierarchical framework comprising seven core constructs of MBIs: Music Features; Person-Specific Music Factors; Engagement Mode; Music Delivery Factors; Social and Environmental Contexts; Music Selection; and Additional Non-Music Components. Across these constructs the taxonomy specifies components, subcomponents, recommended terminology, and definitions to support the conceptualization and description of mechanistic music research.

**Conclusion:**

The MBI Taxonomy aims to improve the consistency and transparency of MBI components, support mechanistic study conceptualization and design, and facilitate replication and cross-study synthesis. As a comprehensive meta-framework, it serves as a foundational tool to help overcome disciplinary silos and foster a more cohesive, rigorous body of mechanistic music research.

## Introduction

Music-Based Interventions (MBIs) are broadly defined as the use of music or music-based experiences to address any dimension of health or human development (Robb et al., 2025a). Music shows clear promise for the treatment and management of several health conditions across the lifespan, including cancer, stroke, dementia, Parkinson's disease, and chronic pain (Cheever et al., 2018). In the context of pain management, MBIs offer distinct advantages: they are low-cost, easy to implement, and have low risk for negative side-effects and complications common to pharmacologic and procedural treatments (Riedl et al., 2025). Similar benefits extend to Alzheimer's disease and related dementias (AD/ADRD), where MBIs are increasingly used as a non-pharmacologic approach to improve behavioral symptoms, cognitive function, and wellbeing among older adults living with the condition (Moreno-Morales et al., 2020).

While the clinical utility of MBIs is evident, researchers have only recently begun to investigate the underlying mechanisms of these treatment effects (e.g., Werner et al., 2023; Pan et al., 2025; Pando-Naude et al., 2019). This emerging line of inquiry remains limited by methodological challenges, and a clear model of underlying mechanisms is still lacking (Bradt et al., 2026). Without this mechanistic knowledge, researchers and clinicians must rely on “trial and error” to improve MBIs for specified health outcomes. Recognizing this critical need, the U.S. National Institutes of Health (NIH) established the U24 Music Research Networks to promote multidisciplinary mechanistic studies of MBIs, with a specific focus on individuals living with AD/ADRD or acute and chronic pain (Burns et al., 2025).

A significant barrier to advancing MBI mechanistic research is the absence of consistent and precise descriptions of study variables, which severely limits our ability to differentiate intervention characteristics responsible for modulating pain perception and AD/ADRD outcomes (Lu et al., 2025; Anderson-Ingstrup and Ridder, 2020; Chen et al., 2022; Martin-Saavedra et al., 2018). Systematic reviews have identified two primary obstacles that limit the validity, replicability, and rigor of MBI research: inadequate reporting and inconsistent terminology. To address reporting quality, the *Reporting Guidelines for Music-Based Interventions* (RG-MBI) were developed to assist authors in describing MBIs with enough detail to support their interpretation and replication (Robb et al., 2025a,b). However, while the RG-MBI standardizes *what* information to report, it does not provide guidance on *terminology* used to precisely label, define, and conceptualize key intervention components.

Despite current guidelines, a critical gap remains: the lack of a shared vocabulary and taxonomic framework to guide the selection and description of MBI components. Because MBI research is conducted by investigators from diverse professional backgrounds—including neuroscientists, musicologists, music therapists, nurses, and physicians—differences in training, musical expertise, and discipline-specific terminology influence how studies are *conceptualized* and the *terms* used to describe the work. Even when similar terms are used, their definitions are often inconsistent (Robb et al., 2018).

This lack of shared meaning diminishes innovation, rigor, and reproducibility in two key ways. First, investigators may limit study conceptualization to music or MBI features most common within their own discipline. Second, the terms used to identify and describe intervention components may lack precision and, perhaps more importantly, shared meaning and clear manualization (Anderson-Ingstrup and Ridder, 2020). While interdisciplinary research is essential for breaking down these silos, a standardized taxonomy of key terms and definitions is needed to improve scientific communication and accelerate discovery (Hodgson, 2019).

To address these limitations and facilitate scientific consensus, the U24 Taxonomy Working Group (WG) was given a two-fold charge: (1) to develop a framework of MBI components and subcomponents to support mechanistic research conceptualization, and (2) to establish a standardized taxonomic framework that includes recommended terms and definitions drawn from across various fields.

The WG focused on identifying components broadly applicable to MBIs, regardless of population, intended mechanisms, or outcomes. This effort included two phases: during Phase I, an Expert Panel developed an initial framework and definitions, and during Phase II a two-part systematic review of MBI and laboratory-based music studies was conducted to validate, interrogate, and refine the framework. Our fundamental intent was to provide a common structure, shared terminology, and consensus definitions to ground future research, support study conceptualization, and facilitate more nuanced descriptions of music and MBI conditions under investigation.

## Method

### Expert panel work group composition

The multidisciplinary Work Group (WG) was comprised of scientists and clinicians from the United States, representing four National Institutes of Health (NIH) U24 Networks (Burns et al., 2025). Members represented a variety of professional disciplines with expertise in music cognition and neuroscience, music therapy, music psychology, music composition, and music performance. The group initiated its work in August 2024. The core WG consisted of seven members (SR, JB, DB, KC, JD, JJ, SRM, JS). During Phase I, this group met twice monthly (for 6 months) to develop the initial taxonomic framework, definitions, and first draft illustration. This preliminary work was then circulated to additional experts for feedback before beginning the next phase. Phase II was co-led by the co-first author (EH), WG Chair (SR), and the medical librarian (MR). EH subsequently managed a team of four graduate music therapy research fellows (LG, SB, JK, MR), who were responsible for data extraction and validation.

### Phase 1: develop initial framework and definitions

Phase I focused on establishing the *intended function* and *structure* of the MBI Taxonomy. The primary function was to create a meta-framework with standardized, well-defined terms to help investigators identify and describe the MBI features under investigation. A hierarchical framework was determined to be the most appropriate classification structure because it organizes related entities into superordinate and subordinate categories based on similarity (Stavri and Michie, 2012), with initial efforts focused on identifying music features and core intervention components. The group worked to identify single, specific, and irreducible components of MBIs—elements that could be used alone or in combination and that may contribute to change in a given outcome (Stavri and Michie, 2012).

From August 2024 to October 2025, the WG used an iterative consensus process to build the initial framework, which involved: (1) establishing categories and subcategories of MBI components and (2) identifying terms and definitions drawn from seminal texts and the extant peer-reviewed literature. This work also yielded an illustrative figure depicting the relationship between music/MBI components and the activation of mind/body systems, to support mechanistic conceptualization (see Supplementary File 1). In November 2024, the drafted Taxonomy (components, terms, and definitions) and figure were circulated to six independent external experts from the fields of pain psychology, neuropsychology, neuroscience, music therapy, and rehabilitation medicine for commentary and suggested edits, which were incorporated before beginning Phase II.

### Phase II: systematic review

The WG, in consultation with our medical librarian co-author (MR), planned and executed a two-part systematic review of MBI and laboratory-based music studies to validate, interrogate, and refine the drafted Taxonomy. Given the intended breadth of the taxonomy across ages, populations, outcomes, and both clinical and laboratory-based research, the group determined that 200 articles (100 clinical studies and 100 lab-based studies) would provide a sufficiently diverse sample to evaluate and refine the taxonomy, with the option to review additional studies if consensus was not reached. Over 4 months (January–May 2025), the data extraction team identified, screened, and extracted data from 200 articles and used a phased, iterative, and consensus-based process to further refine the taxonomy based on published extant literature.

#### Literature search

On January 3, 2025, we conducted systematic searches across four databases: MEDLINE(R) ALL (Ovid), CINAHL Complete (EBSCOhost), Embase (Ovid), and PsycINFO 1887-current (EBSCOhost), with results limited to publications from January 2020–January 2024 and restricted to English language articles. Additionally, we applied filters to retrieve only human studies and excluded review-related studies (systematic reviews, meta-analyses, narrative reviews, and literature reviews).

Two separate search strategies were developed to capture the literature comprehensively. *Search One* focused on MBI clinical trials, combining controlled vocabulary terms (MeSH headings) with natural language keywords to identify experimental and quasi-experimental studies. We applied a study design filter for experimental and quasi-experimental studies to ensure the retrieval of intervention research. *Search Two* focused on lab-based music perception and music neuroscience studies investigating brain mechanisms and psychophysiological responses to music in non-clinical settings. A broad empirical research filter (inclusive of basic research studies) was applied in this second search approach. The search strategies were developed by a librarian (MR), and the pilot searches were peer-reviewed by three experts within our team (see Supplementary Files 2, 3 for the complete search strategies).

**Figure 1:**
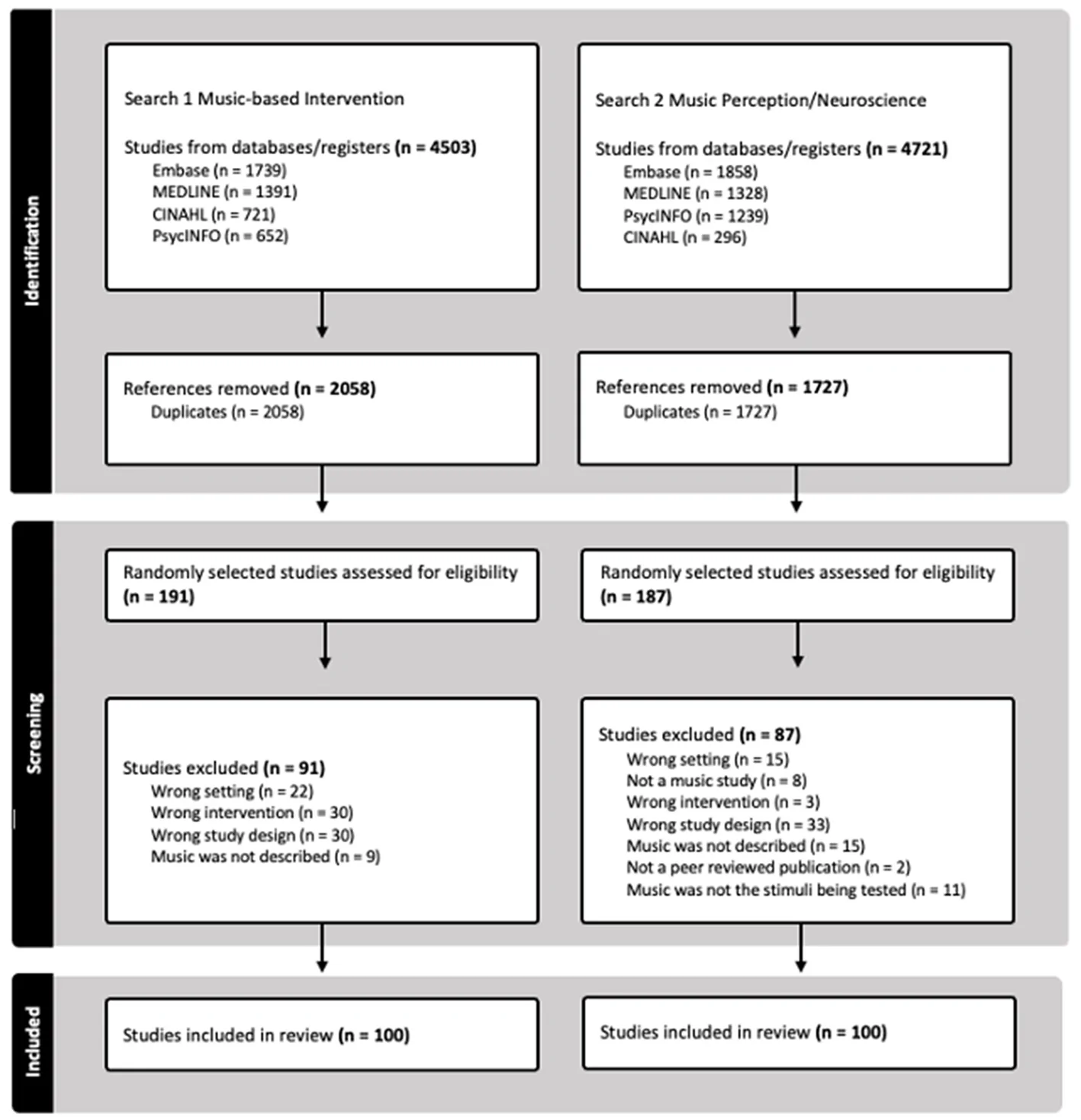
PRISMA diagrams for searches 1 and 2.

All retrieved records were uploaded to Covidence (https://www.covidence.org/) review software for aggregation and deduplication, in two separate reviews. The initial searches identified 2,254 MBI studies and 2,803 lab-based studies. To reach 100 studies that met all inclusion criteria for data extraction, 191 studies were randomly selected, screened, and reviewed. Similarly, for lab-based music studies, 187 randomly selected articles were screened and reviewed to reach 100 studies. See Figure 1 for the PRISMA flow diagram.

#### Data extraction, validation, and iterative revision

To systematically capture and compare the reported components using the Phase I Taxonomy, a custom data extraction template was developed using Covidence (Supplementary File 4). This tool was explicitly designed to mirror the Taxonomy's structure, incorporating all components, subcomponents, and their associated definitions. Five research fellows with expertise in music and MBIs served as Data Extractors (DEs). For each component/subcomponent, DEs followed a two-step process: (1) determine whether the study authors reported information on the component; and (2) if reported, compare the authors' description to the Taxonomy's definition to assess its adequacy. Additionally, DEs documented any novel components not included in the existing Taxonomy, along with the definition or description provided by the study authors. For quality control, all data extractions were completed independently by one DE and subsequently verified by the lead DE for accuracy and completeness.

**Figure 2:**
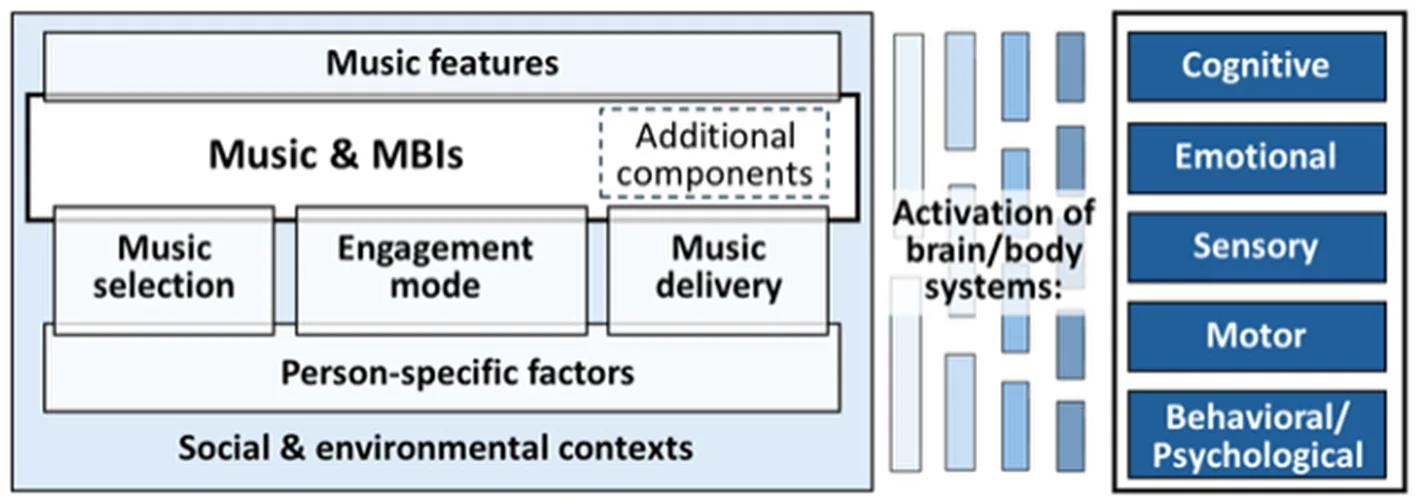
Illustration of the MBI taxonomy.

We used a phased, iterative, and consensus-based process across four rounds of data extraction and consensus meetings to update and validate the Phase I Taxonomy. The first two rounds examined Search One MBI studies, and the two subsequent rounds examined Search Two lab-based studies. Extraction was paused following each round, and the Taxonomy (and thus the extraction template) was revised based on the newly extracted data. Specifically, Round One paused after 70 MBI articles, Round Two after the final 30 MBI articles; Round Three paused after 50 lab-based studies, and Round Four after the final 50 lab-based studies. Following the last round of extraction and review, the Taxonomy was circulated to all WG members and two external reviewers for final revision. In addition, the WG updated the illustrative figure (see Figure 2).

## Results

### MBI taxonomy overview

The resulting MBI Taxonomy includes seven defining constructs of MBIs: Music Features, Person-Specific Factors, Engagement Mode, Music Delivery Factors, Social and Environmental Contexts, Music Selection, and Additional Non-Music Components. For each construct, Tables 1–7 summarize the primary components, sub-components, terms, and definitions (see Supplementary File 5 for a downloadable file of the full Taxonomy). Here we briefly describe each MBI construct, its relative importance to mechanistic research, and the importance of adopting a common nomenclature.

**Table 1:** Music features: components, subcomponents, and definitions.

Musical features		Refers to the compositional, psychoacoustic, and sociocultural elements of music
**Compositional features**		Refers to the five elements of music: melody, harmony, rhythm, color (texture/sound), form as well as genre and tonality.
**Component**	**Subcomponent**	**Definition**
**Melody**		A consecutive series of sounds (pitches) and silences (rests) arranged in time (rhythm) (Latham, 2002).
**Harmony**		Two or more pitches sounding simultaneously to produce chords that may be perceived as either stable (consonant) or unstable (dissonant). Successive chords produce a harmonic progression (Latham, 2002).
	Consonant harmony	A chord (simultaneous sounding of two or more pitches) that is heard as being stable, pleasing, or complete within the tonal/modal norms of a particular culture (Latham, 2002).
	Dissonant harmony	A chord that is heard as being unstable, tense, transitional, or in need of resolution within the tonal/modal norms of a particular culture (Latham, 2002).
**Rhythm**		The pattern of musical elements in time, including tempo, meter, syncopation, and silence (rests) (Latham, 2002; LaRue, 1992).
	Meter	Pattern of strong and weak beats (or pulses) which organize a piece of music (Latham, 2002).
	Syncopation	Compositional device that destabilizes the expected or established rhythmic pattern (meter) and creates a sense of anticipation for the accented beat (e.g., through displacing accent on weak beat, resting on strong beat).
	Tempo	The speed at which a piece of music is performed (often reported as beats-per-minute) (Latham, 2002).
**Sound**		Aspects of sound include timbre, texture, dynamics, and range/tessitura (Latham, 2002).
	Timbre (color)	The unique sound produced by a particular instrument or voice or combination of instruments/voices (Kimi, 2019).
	Texture	The overall quality or complexity of a piece of music, based on how each music feature manifests and relates to other features. Considerations include but are not limited to: presence/absence of accompaniment and number of melodic lines (e.g., monophony vs. polyphony), presence/absence of lyrics, and vertical spacing of chords (i.e., density) (Barbacane, 2019).
	Dynamics	Perceived variations in loudness and softness of music, and how much contrast there is between the loudest and the quietest sounds. This is not the same thing as decibel volume or acoustic loudness (see Table 1. Psychoacoustic Features).
	Range/Tessitura		
	*Range*	Distance between the highest and lowest pitches.
	*Tessitura*	The portion of the full range most used or emphasized by an instrument or vocalist in a piece of music.
**Silence**		The intentional or unintentional absence of music or sound.
**Form**		The application and organization of all musical elements in a piece to create a sense of direction and shape through the use of repetition, contrast, and development (Latham, 2002; LaRue, 1992).
**Genre (compositional)**		Constructed categories of music that share a distinct set of musical characteristics or features, such as a particular rhythmic pattern or instrumentation. Genres may also be constructed based on sociocultural, historical, or artistic contexts (see Table 1. Sociocultural Features) (Fabbri, 1982).
**Tonality (compositional)**		The relative organization of all the tones and harmonies of a piece of music and the relationships between them, often described in relation to a prominent, recurring note (tonic) (Hyer, 2012).
**Psychoacoustic features**		Refers to the physical parameters of sound, including pitch, loudness, sharpness, tonality, duration, and roughness.
**Component**	**Subcomponent**	
**Pitch**		Subjective perception of a sound's position within the musical scale (i.e., high or low), determined by the objective frequency of the sound (i.e., rate of oscillation per second) (reported in hertz) (Truax, 1999).
**Loudness (amplitude)**		Subjective impression of the intensity of a sound (may be reported in decibels) (Truax, 1999; Crombie, 1986).
**Sharpness**		Subjective perception of sounds with an emphasis on high frequencies as being harsh or piercing (e.g., cymbal crash).
**Booming**		Subjective perception of sounds with an emphasis on low frequencies as being resonant and impactful (e.g., bass drum).
**Tonality**		Indicator of tones that the ear perceives and draws out of a mass of sound (Redon, 2022).
**Duration**		The length of time of a note or full piece of music is played.
**Roughness**		Subjective perception of sounds with an emphasis on rapid pitch fluctuations as being grating, unpleasant, or annoying (e.g., a poorly tuned string instrument or a wide vocal vibrato) (Daniel, 2008).
**Salience**		The extent to which a sound stands out from other sound in the acoustic environment (Huang and Elhilali, 2017).
**Sociocultural features**		Refers to aspects of music (including genre) that reflect cultural identity, societal values, and social functions, including but not limited to musical traditions/heritage, rituals, ceremonies, celebrations, healing practices, and political protest/activism.

#### Music features

Music features refer to the compositional, psychoacoustic, and sociocultural elements of music (Table 1). These features are organized into three primary categories. *Compositional Features* include seven foundational elements of music, including melody, harmony, rhythm, sound, silence, form, genre, and tonality. *Psychoacoustic Features* refer to the physical parameters of sound, such as pitch (frequency), loudness (amplitude), sharpness, tonality, duration, and roughness that influence how music is processed and perceived by the human auditory system. *Sociocultural Features* reflect assigned or inferred cultural identity, societal values, and social functions.

As a central defining component of all MBIs, music is an inherently complex stimulus. This complexity presents a significant challenge for mechanistic research aimed at discerning which specific features (or combination of features) are responsible for observed changes in a target outcome. This challenge is particularly evident when bridging the gap between laboratory and clinical research. For example, a laboratory study might isolate specific psychoacoustic features to observe their effects under highly controlled conditions; however, these findings may lack ecological validity by omitting compositional or sociocultural features that are central to real-world music experiences. In contrast, clinical studies often use music experiences that layer these features, making it difficult to isolate the precise “active ingredient” responsible for change. Without a shared nomenclature to describe the full array of music features, it remains difficult to synthesize findings across lab and clinical contexts to determine how both individual and integrated music features drive psychological and physiological responses (Saskovets et al., 2025).

#### Person-specific music factors

Person-specific music factors refer to multidimensional factors that may be unique to an individual and may influence their interaction with and response to music. These factors include prior musical training or exposure, music-evoked responses, music preference, and familiarity (Table 2). They are shaped by a broad range of internal and external dimensions, such as environment, emotion, cognition, personality, identity, and the auditory system (e.g., ear morphology, sound sensitivity) (Rentfrow and Gosling, 2003; Thompson et al., 2023; Li et al., 2025; MacDonald and Saarikallio, 2022; Hansen et al., 2025). Furthermore, this construct encompasses an individual's personhood, beliefs, values, and behaviors, alongside their specific music preferences and degree of familiarity gained through previous exposure (Greasley and Lamont, 2016; Mehdizadeh and Leslie, 2023; Rentfrow et al., 2011; Jagiello and Pomper, 2019; King and Prior, 2016; Meyer, 1903; van den Bosch et al., 2013; Zatorre, 2015).

**Table 2:** Person-specific music factors.

Person-specific music factors		Refers to factors that may be unique to an individual. Person-specific factors are influenced by a range of dimensions, including but not limited to life events, emotion, cognition, personality, self-identity, beliefs, preferences, and behaviors.
**Component**	**Subcomponent**	**Definition**
**Music preference**		A person's liking for one piece of music, music genre, artist, or style as compared to another at a given point in time (Greasley and Lamont, 2016; Mehdizadeh and Leslie, 2023; Rentfrow et al., 2011).
**Familiarity with music**		Feeling of being acquainted with a piece of music due to previous exposure to the piece or similar pieces (Jagiello and Pomper, 2019; King and Prior, 2016; Meyer, 1903).
**Prior musical training/experience**		Previous formal/informal music training or sustained engagement in vocal or instrumental music making (Kraus and Chandrasekaran, 2010).
**Music-evoked responses**		Refers to autobiographical, emotional, physical, and physiological responses evoked by music.
	Autobiographical memories/meaning	Memories and/or meaning evoked when hearing a piece of music from one's past (Janata et al., 2007).
	Musical valence and arousal	Refers to the propensity of music to evoke a range of affective and emotional responses which may be associated with varying levels of physiological activation (i.e., arousal) (Warrenburg, 2020).
	Spontaneous movement	Unprompted movement that may occur when listening to or experiencing music.
	Auditory processing related factors	Refers to conditions impacting the central nervous or auditory systems which affect auditory stimulus discrimination, auditory stimulus tolerance, and/or how the brain processes sound information (e.g., autism, acquired brain injury, or hearing impairment).

Prior music training or music-making experiences across the life course also contribute to our responses to music at a given time (Golubovikj et al., 2025). For example, autobiographical memories are often evoked when hearing a piece of music from one's past (Janata et al., 2007); these music-evoked memories often are meaningful to the individual (Belfi et al., 2016). The preference for or liking of specific aspects of music can also change across the life course (Golubovikj et al., 2025). At a given time, an individual may prefer or like one piece of music, music genre, artist, or style compared to another time (Greasley and Lamont, 2016; Mehdizadeh and Leslie, 2023; Rentfrow et al., 2011). Familiarity with the music is also a person-centered aspect, which can vary from being unfamiliar to highly familiar (Jagiello and Pomper, 2019; King and Prior, 2016; Meyer, 1903). Music can also evoke a range of positive and negative emotion/affective responses, which may be associated with high or low levels of arousal (Warrenburg, 2020). Interestingly, some individuals do not find music pleasurable, a phenomenon known as musical anhedonia (Martínez-Molina et al., 2016). Music often evokes movement, such as tapping a foot, clapping, and swaying. Listening to music activates brain regions that control movement and often result in spontaneous movements that are synchronized with the music (Hurley et al., 2014). These movements evoked by music are often automatic and spontaneous and can often feel good, such as when we are in the “groove” (Janata et al., 2012). Together, these factors form the personal lens through which music is processed and assigned meaning.

A significant barrier to advancing mechanistic research is the lexical imprecision surrounding how person-specific factors are accounted for in study designs. Currently, a disconnect exists between how music is selected and how its effects are interpreted. In many studies, investigators select the music without assessing how well these stimuli align with person-specific factors (Menza et al., 2024). For example, researchers often select music based on standardized genres common to their own culture or country (e.g., Thoma et al., 2013). When music is participant-selected, the specific process for that selection is also frequently under-described, making replication impossible (Maidhof et al., 2025; Ballmann et al., 2025). The few studies that have compared the effects of researcher- and participant-selected music suggest that the source of music selection can affect the outcomes (Maidhof et al., 2023; Groarke et al., 2020).

An absence of a shared framework hinders discovery by failing to standardize the “subjective” as a measurable variable. For example, researchers have often labeled music as “relaxing” based on its acoustic or compositional features. Yet, the experience of relaxation is highly individual and contingent on preference and familiarity, both of which are known to modulate neural reward systems and physiological pleasure (Zatorre, 2015; Gold et al., 2023). Without consensus definitions to label these person-specific variables, it is difficult to differentiate whether a therapeutic effect is driven by the music's structural features, the participant's personal history with the music, or both.

#### Engagement mode

Engagement mode refers to the specific ways in which a person interacts with music, ranging from receptive listening to active creation. This construct encompasses various forms of interaction, including listening to prerecorded or live music, recreating music thorough singing or playing instruments, composing, improvising, learning music skills, and experiencing music through audio- or vibro-tactile stimulation (Table 3). Engagement modes differ in their demands and sensory inputs, varying in levels of cognitive load, motor activity, creativity, and locus of control. For example, a receptive interaction like listening through headphones offers a different sensory experience than an active interaction like songwriting or instrumental improvisation, which may involve higher degrees of risk-taking or a sense of accomplishment.

**Table 3:** Engagement mode.

Engagement mode	Refers to how a person engages with music
**Component**	**Definition**
**Listening to music**	Listening to live or recorded music (Reybrouck et al., 2021).
**Composing music**	Writing songs, lyrics, or instrumental pieces, or creating any kind of musical product (Bruscia, 2014).
**Re-Creating music**	Rendering, reproducing, realizing, or interpreting all or any part of an existing musical work (Bruscia, 2014). Re-creating can be instrumental, vocal, or computer generated.
**Improvising music**	Extemporaneously creating music using instruments, voices, or other sound sources (e.g., body percussion; computer-generated sounds).
**Music instruction**	Developing musical skills, knowledge, and understanding through a variety of methods, techniques, and approaches (Lehmberg, 2012).
**Audio- or vibro-tactile stimulation**	Providing tactile sensations that complement or enhance the auditory music listening experience. Often achieved through devices that convert musical sound into vibrations that can be felt on the skin (Siedenburg et al., 2024).

To date, research that examines mechanisms underlying the health benefits of music has primarily focused on listening to prerecorded music in laboratory settings (Bradt et al., 2026). Many of these studies use prerecorded music to standardize music stimuli and isolate a candidate mechanistic variable. In contrast, while tailored live music is a common feature of clinical practice, research examining how such personalized interventions work mechanistically is less represented in the literature. For example, a therapist might adjust the tempo of live music to promote relaxation or delay chord resolutions to prompt a response from a participant with cognitive deficits. How participants are instructed to listen to the music—such as focusing on the lyrics or playing music in the background—may also have a differential effect. While lab studies of prerecorded music listening have provided valuable insights, this represents only one facet of musical engagement. It is equally important to investigate other engagement modes to understand their unique effects and underlying mechanisms. In particular, active and social forms of music-making may engage entirely different neural, physiological, and psychological pathways, offering distinct benefits that remain largely unexplored.

#### Music delivery factors

Music delivery factors refer to the music delivery schedule, the delivery method, and the qualifications of the facilitator (Table 4). These elements define the temporal dose of exposure, the sensory and perceptual qualities of the sound signal, and the relational or adaptive capacities of the intervention. Specifically, the delivery schedule accounts for frequency and duration, while the delivery method describes how the sound is physically transmitted, and facilitator qualifications identify the expertise that influences the degree of real-time clinical adaptation possible during an intervention.

**Table 4:** Music delivery factors.

Music delivery factors	Refers to the music delivery schedule, delivery method, and facilitator qualifications
**Component**	**Definition**
Delivery schedule	Information about participants' exposure to a music experience (or music stimulus). This might include: (1) number of sessions (exposures); (2) length of each session (exposure) (e.g., minutes/h); (3) frequency of sessions—how often the exposure happens over a specified period of time (e.g., 3x/week); (4) time interval between sessions—when applicable, indicate time between sessions (exposures); and (5) duration of the program (or music exposures) over time (e.g., over 4 weeks). Delivery schedule might also include information about practice, experiences, or tasks that are assigned to participants between intervention or study sessions (Robb et al., 2025b).
Delivery methods	Refers to the ways in which music is provided to or created with participants, (e.g., via live, recorded, or computer-generated means; via headphones or free field), including, if relevant, whether and how the music is adapted in the moment to intentionally influence participants' physiological or psychological responses.
Facilitator qualifications	Refers to the professional background, credentials, training, and/or experience of the facilitator(s) of MBIs.

Mechanistic work supports the multidimensional influence of music delivery factors. However, a lack of standardized terminology has often led to these elements being dismissed as procedural details. This oversight hinders our ability to isolate how the *way* music is delivered influences neurophysiological and psychosocial outcomes. For example, research has demonstrated that virtual live music delivered through videoconferencing preserved interpersonal presence and allowed for real-time adaptation of musical elements, which influenced anxiety and autonomic indicators in ways that differed from prerecorded audio (Bruder et al., 2024). Similarly, the use of free-field delivery in the Intensive Care Unit interacted with ambient noise, spatial orientation, and staff activity, which shaped attentional demands, perceptual load, and the integration of audio cues in a high-acuity environment (Kleinpell et al., 2024). Recent EEG-based work further demonstrates how delivery method and schedule influence neurophysiological markers (Moe et al., 2025). Distinct 3-min live and recorded music conditions produced different patterns in entropy, variability, and complexity metrics, suggesting that live music and recorded music each engage neural systems differently, even when compositional content is similar. These findings highlight that delivery method is not merely a procedural detail, but rather an important variable that affects entrainment, emotional salience, auditory prediction, social co-regulation, and brain network dynamics (Gururaja et al., 2022; Bruder et al., 2023).

#### Social and environmental contexts

Social and environmental contexts refer to the relational and physical circumstances under which a music experience occurs (Table 5). This includes the number of people involved, their specific roles (e.g., client, family, or peers), and the nature of the interaction, which may be self-directed or guided by a therapist or facilitator. Music experiences are inherently varied across these dimensions. Music can be experienced or created in isolation, with another person, or among larger groups. Furthermore, these experiences are often shaped by the physical location—such as a home, laboratory, public space, or virtual environment—and the presence of ambient sound. Together, these factors may help to define the lifeworld of the participant, encompassing both the immediate sensory environment and the social dynamics that influence how music is received and assigned meaning (Abbas et al., 2024).

**Table 5:** Social and environmental contexts.

Social and environmental contexts		Refers to social, relational, and environmental circumstances under which a music experience (or stimulus) occurs, including the number of people involved, their role(s), and the surroundings. Music can be experienced or created alone, with another person, or in larger groups (Perkins et al., 2020)
**Component**	**Subcomponent**	**Definition**
**Relational context**		Refers to the number of people involved in a music experience and whether the experience is facilitated.
	Number of people	Refers to the number of people involved in the music experience (alone, with another person, or in a group).
	Facilitated or not facilitated	Refers to whether a music-based experience was guided by a facilitator.
**Environmental context**		Refers to the surroundings and conditions in which a music experience (or stimulus) occurs, such as location (e.g., home, lab, public space, virtual), presence of people other than participants/facilitators, or the presence of ambient sound.

A significant barrier to advancing mechanistic research is the conceptual blurring of “social” and “environmental” factors, which are often treated as implicit or interchangeable. The lack of clear terminology makes it difficult to differentiate the psychological and neural mechanisms of social connection from the physical impact of the environment. For example, while researchers may use general terms like “social setting,” the specific relational mechanisms that drive wellbeing—such as those distinguished in studies on social connection (Delgado et al., 2023) or music therapy for dementia (Reschke-Hernández et al., 2023)—require precise labeling to be replicated. Without consensus definitions, investigators may overlook important acoustic or relational features that independently contribute to music's health benefits.

Furthermore, adopting a guiding taxonomic framework is essential to address the lack of diversity in MBI research. Study populations in music-based research often lack sociodemographic, geographic, and economic diversity, typically favoring well-resourced environments (Schimmelpfennig et al., 2025; Bradt et al., 2021; Kang et al., 2025). This narrow focus limits the generalizability of mechanistic findings. Without a common taxonomy that recognizes the importance of diverse contexts, researchers may overlook how varying cultural and social factors modulate physiological and psychosocial responses to music.

#### Music selection

Music selection refers to the person(s) responsible for choosing the music in a research study and the specific methods used to make those choices (Table 6). Those responsible for selection generally fall into three categories: investigators, therapists or facilitators, and participants or their family members. The selection process is further classified into four categories: pre-identified parameters (such as specific tempo algorithms or AI generation), participant preference, clinical assessment, and collaborative decision-making.

**Table 6:** Music selection.

Music selection	Refers to the person(s) who select the music in a music research study and the process for making selections (Robb et al., 2025a)
**Component**	**Definition**
**Person(s) selecting the music**	The individuals who select music fall into three general categories: (1) the investigator(s), (2) the therapist(s) or facilitator(s), and (3) the participant(s), their caregiver(s), or family member(s). Selection may involve individuals from one or more than one of these categories.
**Process for selecting the music**	Process for Selecting the Music—The processes for selecting music used in a music research study fall into four general categories: (1) pre-identified parameters (e.g., specific music element such as tempo, algorithms or AI generation), (2) participant preference, (3) assessment, and/or (4) collaborative decision making (Loewy, 2000; Loewy et al., 2021; Raglio et al., 2020; Xu et al., 2021).

Both the selector and the selection process are important considerations in mechanistic research because they influence the participant's degree of agency. For example, an investigation by Howlin and Rooney showed that increased levels of perceived choice (e.g., choosing from four songs vs. two songs vs. no choice) were associated with higher pain tolerance, even after accounting for music enjoyment (Howlin and Rooney, 2021).

Finally, a recent systematic review and meta-analysis of nine MBI randomized controlled trials concluded that interventions were most effective when participants selected their own music (Chen et al., 2025). However, the authors only described who selected the music and did not include information about the selection process. Given these findings and the significant role that participant autonomy plays in modulating therapeutic outcomes, it is important that investigators consider both the person(s) selecting the music and the process for making those selections.

#### Additional non-music components

Additional non-music components refer to components supported by or used in combination with music (Table 7). These components often include body movement, mental imagery, relaxation techniques (such as mindfulness or breathwork), and other arts media. Further adjuncts include spiritual practice, discussion, biofeedback, and human touch. These non-music elements are frequently integrated into MBIs to deepen engagement or target specific physiological or psychological systems.

**Table 7:** Additional non-music components.

Additional non-music components	Refers to components supported by or used in combination with music (Lepping et al., 2024; Robb et al., 2018; Rodwin et al., 2023)
**Component**	**Definition**
**Body movement**	Prompted change(s) in the positioning of a body part(s) relative to the entire body. This can include (but is not limited to) a wide range of voluntary gross and fine motor movement such as tapping, arm raises, swaying, walking, or the use of movement to express internal states and emotions (Cruz-Garza et al., 2014; Virameteekul and Bhidayasiri, 2022; Gonzalez-Sanchez et al., 2018).
**Imagery**	Mental images experienced spontaneously or in response to suggestion or some other form of sensory stimuli.
**Relaxation**	Activities or practices intended to relieve tension. These may include, but are not limited to, mindfulness, meditation, and breathwork.
**Other arts media**	Refers to arts media other than music. These may include, but are not limited to, storytelling, drama, visual arts, dance, or virtual reality.
**Spiritual practice**	Activities and rituals used to deepen one's relationship or sense of connection with nature, the sacred/divine, a higher power(s), and/or the universe. Practices may include, but are not limited to, prayer, time in nature, meditation, and worship.
**Analysis/discussion**	The act or process of discussing or examining something to gain clarity or insight, reach a decision, and/or exchange ideas.
**Biofeedback**	The process of training someone to acquire voluntary control of a typically involuntary physiological or brain function through closely monitoring that function.
**Human touch**	The act or process of providing wanted, supportive, intentional forms of human touch, including but not limited to massage, comfort positioning, and holding a person.

The integration of non-music adjuncts complicates the identification of specific intervention features responsible for change. Because these components are often integrated seamlessly with music, it can be difficult to isolate whether a therapeutic effect is produced by the music experience alone or by the synergistic influence of combined elements. For example, the use of mental imagery—whether experienced spontaneously or through directed suggestion—activates neural mechanisms distinct from those involved in music listening alone (Lee et al., 2016). Similarly, the inclusion of body movement, such as motoric entrainment or rhythmic tapping, engages sensorimotor pathways that are not activated during passive listening (Vuilleumier and Trost, 2015). A shared nomenclature to distinguish these adjuncts can assist researchers in considering the specific contributions of the music vs. the non-music components when designing their studies.

The need for precise categorization is further underscored by evidence that subtle variations in how these adjuncts are delivered can lead to distinct neurophysiological outcomes. In relaxation and mindfulness research, for instance, a body scan with instructions that emphasize “awareness” activates different neural pathways than a body scan with “physical relaxation” instructions (Sevinc et al., 2018). Such nuances demonstrate that non-music components are not mere fillers but are active variables that must be well defined to ensure reproducibility. By standardizing the language for these adjuncts, our framework provides a systematic way to account for the multimodal complexity inherent in many music-based interventions. This descriptive precision is essential for understanding how these additional factors influence the outcomes of mechanistic trials and for predicting how music interacts with other therapeutic modalities to drive health change.

## Discussion

Taxonomies are systems that classify and structure information into hierarchical or thematic categories based on shared characteristics, establishing a common language to name, define, and describe the world (Yazdani et al., 2021). They play an important role in scientific inquiry by improving search accuracy, standardizing terminology, supporting study conceptualization, and enabling meaningful synthesis across growing evidence bases. In health research, taxonomies are particularly valuable for organizing complex, multi-component interventions and clarifying how intervention features are described, compared, and interpreted across studies.

Within the field of arts and health, several influential frameworks have been developed to support these goals. Notably, the *INNATE Framework* provides a comprehensive mapping of 139 potential “active ingredients” across project, people, and contextual domains, serving as a shared dictionary to facilitate dialogue across disciplines and art forms (Warran et al., 2022). Similarly, the *Taxonomy of Arts Interventions for People with Dementia* specifies 12 dimensions to classify creative activities and improve communication, evaluation, and evidence mapping within a specific clinical population (Cousins et al., 2020). These frameworks have advanced conceptual clarity across the broader arts-in-health literature, particularly by highlighting the complexity and interdependence of intervention components.

However, the breadth that gives multimodal frameworks their inclusivity also limits their precision for investigators designing studies within a single artistic modality. By necessity, cross-art frameworks prioritize generalizability over specificity and avoid replacing vocabularies unique to individual art forms (Warran et al., 2022). As a result, they are less suited to defining, with sufficient specificity, the musical features and intervention components hypothesized to drive specific neurobiological, psychological, or behavioral mechanisms.

More narrowly focused tools also exist within music and health research. The *Music and Health Institute Taxonomy* (Music and Health Institute, n.d.) was developed primarily as a records-management and indexing system to improve discovery and retrieval of music-related research. Similarly, the *Music in Therapeutic Imagery Taxonomy* was designed to support Guided Imagery and Music practitioners in selecting music based on musical-psychological characteristics aligned with therapeutic goals (Wärja and Bonde, 2014). Although valuable within their respective domains, these tools were not designed to support systematic mechanistic inquiry across music-based intervention research.

The MBI Taxonomy was designed to address this gap. It distinguishes itself as the first music-specific taxonomy of its kind, providing nuanced terms and definitions that enable greater transparency and standardization than is possible in multimodal frameworks. Focusing exclusively on a single modality to identify music-specific features, it offers a well-defined starting point for investigators designing mechanistic trials of music interventions.

Recommended by the *NIH Music Research Network Taxonomy Working Group*, the MBI Taxonomy was designed to support the conceptualization, description, and synthesis of mechanistic research. It facilitates cross-study comparison by offering a recommended list of terms and definitions to describe MBI features and components. The taxonomy comprises seven overarching constructs: Music Features, Person-Specific Factors, Engagement Mode, Music Delivery Factor, Social and Environmental Contexts, Music Selection, and Additional Non-Music Features. Within each construct, the taxonomy further delineates primary components and subcomponents. Importantly, this framework was designed to address established barriers to advancing our mechanistic understanding of music interventions while fostering a more cohesive body of interdisciplinary research.

### Implications for mechanistic research

The primary contribution of the MBI Taxonomy lies in its ability to strengthen mechanistic research by improving conceptual rigor at the study-design stage. Mechanistic investigations require clear articulation of the components under study and explicit justification for why particular features are hypothesized to influence specific brain, body, or behavioral processes. By providing a structured vocabulary and music-specific framework, the taxonomy helps investigators to specify *what* aspects of music and music-based interventions are being manipulated, compared, or controlled.

Importantly, the taxonomy does not identify mechanisms of action or specify how components should be measured or tested. Rather, it establishes the foundational conditions necessary for mechanistic inquiry by clarifying intervention features in a consistent and transparent manner. This distinction is particularly important for bridging laboratory-based music research with clinical MBI studies, where historically divergent terminology and incomplete intervention description have limited interpretation, replication, and synthesis.

### Implications for reporting, replication, and evidence synthesis

The MBI Taxonomy also addresses persistent challenges in reporting quality and reproducibility. While existing reporting guidelines specify *what* information should be included in intervention descriptions, they do not address variation in the terminology used to describe music-based components (Robb et al., 2025a). By offering recommended terms and definitions, the taxonomy complements existing reporting standards and improves transparency. Over time, consistent adoption of this framework may enable more meaningful meta-analyses, secondary analyses, and cross-study syntheses within mechanistic research.

### Limitations

While the MBI Taxonomy provides a rigorous meta-framework for standardizing terminology, several limitations warrant consideration. First, the development process involved geographic and linguistic constraints, as the systematic review was limited to English-language studies and the expert panel was comprised solely of United States-based representation. These factors may introduce bias and limit the identification of essential intervention components, the selection of specific terms, and the universality of the provided definitions. Additionally, the framework's validity and utility must be formally evaluated over time through broader application. The MBI Taxonomy is not a static document but rather a living framework that will require ongoing research and refinement to substantiate its utility as scientific advancements continue.

### Conclusion

The MBI Taxonomy offers a comprehensive meta-framework for conceptualizing the diverse range of music and MBI features that may contribute to brain-body activation (Figure 2). By providing a standardized nomenclature, it aims to improve the consistency and transparency of intervention descriptions, allowing researchers to more effectively account for how musical features interact with person-specific factors and social-environmental contexts. Ultimately, this framework serves as a foundational tool to overcome disciplinary silos and foster a more cohesive, rigorous body of mechanistic music research.

## Data Availability

The original contributions presented in the study are included in the article/Supplementary material, further inquiries can be directed to the corresponding author/s.
