# Supplementary material for: A taxonomic framework of music-based intervention components to guide research: recommendations from the NIH U24 music research networks
Source: Front Psychol. 2026 Sep 9;17:1876240. doi: 10.3389/fpsyg.2026.1876240 (PMC13597745; doi:10.3389/fpsyg.2026.1876240)
Supplement: Supplementary file 1 [file Data_Sheet_1.pdf]

Supplemental File 1.

Initial Illustrative Figure

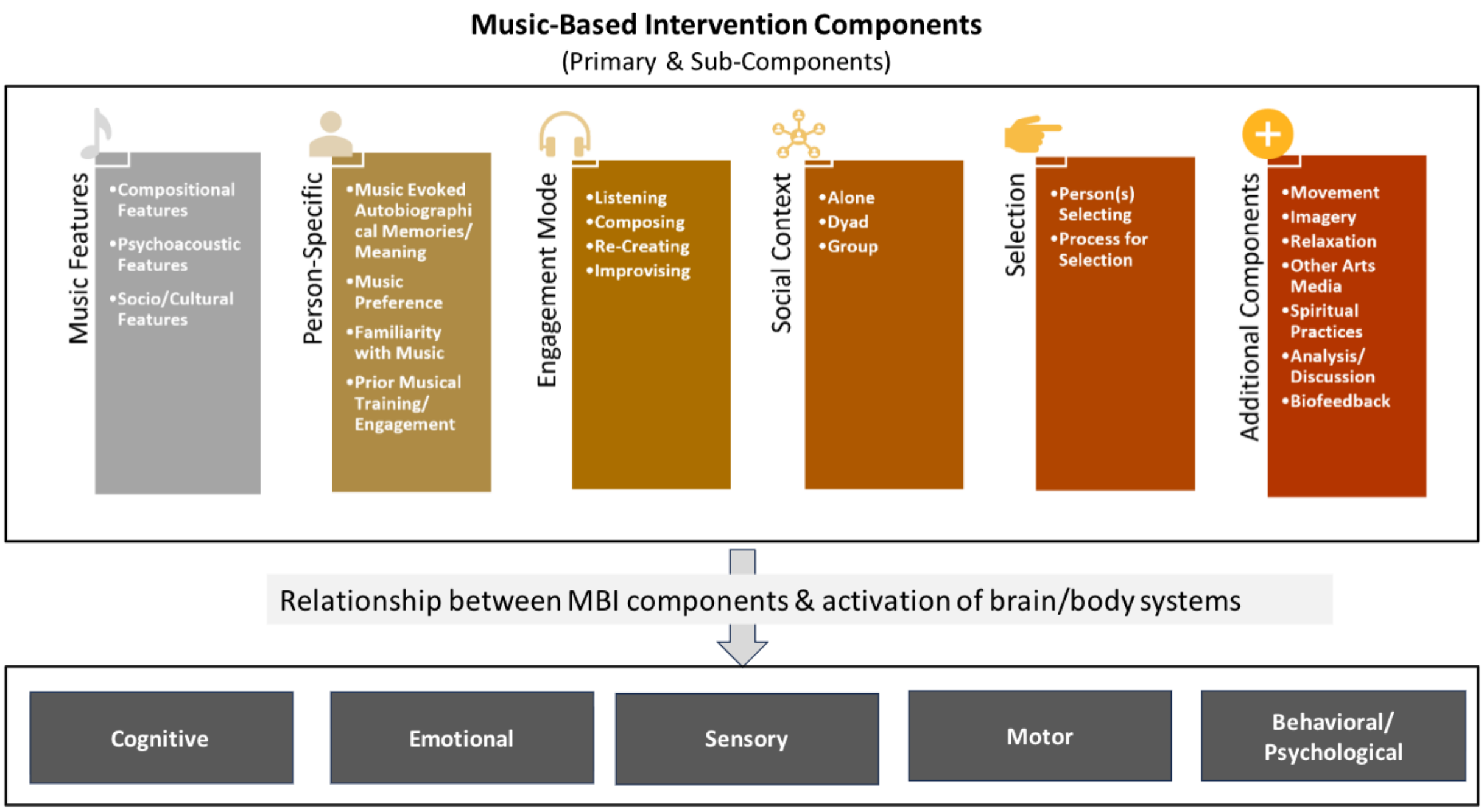

Supplemental File 2

Search 1: Music-based Interventions
Search strategy

MEDLINE(R) ALL(Ovid) (Run on January 3, 2025, 1391 records)

1 "Music Therapy"/ 4748
2 music/px [Psychology subheading] 2563
3 (Music/ or Singing/) and (Psychotherapy/ or Rehabilitation/ or Therapeutics/ or "Imagery, Psychotherapy"/ or "Sensory Art Therapies"/ or "Acoustic Stimulation"/ or "therapy".fs. or "rehabilitation".fs.) 4213
4 (music* and (imagery or intervention* or making or medicine or therap* or treatment* or rehabilitation)).ti,kf. 4897
5 (music* adj3 (imagery or intervention* or making or medicine or therap* or treatment* or rehabilitation)).ab. 5214
6 musicotherapy.ti,ab,kf. 19
7 ((music* or singing or singer or singers or song or songs or vocal or vocalization) and (psychotherap* or "sensory therap*" or "acoustic stimulation")).ti,ab,kf. 601
8 (music* adj3 (activit* or composition or instrument* or improvisation or listen* or performance)).ti,ab,kf. 6837
9 ((chant or chants or songwriting or song-writing or "rhythmic auditory stimulation" or melody or melodies or lullaby or lullabies or "Hip Hop" or (Rock adj2 Roll) or (music adj2 (ambient or classic* or ethnic or folk or jazz or meditative or metal or pop or popular or religious or rock or sacred or spiritual or symphon* traditional))) and (imagery or intervention* or therap* or treatment* or rehabilitation)).ti,ab,kf. 1189
10 or/1-9 18114
11 ("Randomized Controlled Trial" or "Controlled Clinical Trial" or "Pragmatic Clinical Trial" or "Equivalence Trial" or "Clinical Trial, Phase III").pt. 725532
12 "Randomized Controlled Trial"/ 629334
13 exp "Randomized Controlled Trials as Topic"/ 181332
14 exp "Controlled Clinical Trials as Topic"/ 187177
15 "Random Allocation"/ 107967
16 "Double-Blind Method"/ 182109
17 "Single-Blind Method"/ 34418
18 Placebos/ 36032
19 "Control Groups"/ 2140
20 (random* or sham or placebo*).ti,ab,hw,kf. 1981274
21 ((singl* or doubl*) adj (blind* or dumm* or mask*)).ti,ab,hw,kf. 281754
22 ((tripl* or trebl*) adj (blind* or dumm* or mask*)).ti,ab,hw,kf. 2004
23 (control* adj3 (study or studies or trial* or group*)).ti,ab,kf. 1352550
24 (Nonrandom* or non random* or non-random* or quasi-random* or quasirandom*).ti,ab,hw,kf. 60341
25 allocated.ti,ab,hw. 92903
26 ((open label or open-label) adj5 (study or studies or trial*)).ti,ab,hw,kf. 50118
27 ((equivalence or superiority or non-inferiority or noninferiority) adj3 (study or studies or trial*)).ti,ab,hw,kf. 14409
28 (pragmatic study or pragmatic studies).ti,ab,hw,kf. 693

29 ((pragmatic or practical) adj3 trial*).ti,ab,hw,kf. 9042
30 ((quasiexperimental or quasi-experimental) adj3 (study or studies or trial*)).ti,ab,hw,kf. 14924
31 (phase adj3 (III or "3") adj3 (study or studies or trial*)).ti,hw,kf. 38516
32 or/11-31 2833725
33 10 and 32 4406
34 33 and 2020:2024.(yr). 1828
35 34 and English.lg. 1810
36 35 not (exp animals/ not exp humans/) 1793
37 36 not ((review or "systematic review" or "meta-analysis").pt. or "review literature as topic"/ or "systematic reviews as topic"/ or "Meta-Analysis as Topic"/ or ("rapid review" or "systematic review" or "scoping review" or "umbrella review" or "meta analy*" or "metanaly*" or "metaanaly*" or "met analy*" or "narrative review" or "literature review").ti.) 1391

Embase (Ovid) (Run on January 3, 2025, 1739 records)

1 *"music therapy"/ 5949
2 music/ and psychology/ 1134
3 (music/ or singing/) and (psychotherapy/ or rehabilitation/ or therapy/ or "guided imagery"/ or "sensory integration therapy"/ or "auditory stimulation"/ or "th".fs. or "rh".fs.) 5152
4 (music* and (imagery or intervention* or making or medicine or therap* or treatment* or rehabilitation)).ti,kf. 6322
5 (music* adj3 (imagery or intervention* or making or medicine or therap* or treatment* or rehabilitation)).ab. 7444
6 musicotherapy.ti,ab,kf. 83
7 ((music* or singing or singer or singers or song or songs or vocal or vocalization) and (psychotherap* or "sensory therap*" or "acoustic stimulation")).ti,ab,kf. 976
8 (music* adj3 (activit* or composition or instrument* or improvisation or listen* or performance)).ti,ab,kf. 8120
9 ((chant or chants or songwriting or song-writing or "rhythmic auditory stimulation" or melody or melodies or lullaby or lullabies or "Hip Hop" or (Rock adj2 Roll) or (music adj2 (ambient or classic* or ethnic or folk or jazz or meditative or metal or pop or popular or religious or rock or sacred or spiritual or symphon* traditional))) and (imagery or intervention* or therap* or treatment* or rehabilitation)).ti,ab,kf. 1974
10 or/1-9 21707
11 limit 10 to (randomized controlled trial or controlled clinical trial or phase 3 clinical trial) 2579
12 "randomized controlled trial"/ 862540
13 exp "randomized controlled trial (topic)"/ 288039
14 "controlled clinical trial"/ or "controlled clinical trial (topic)"/ 488432
15 randomization/ 100514
16 "double blind procedure"/ 227846
17 "single blind procedure"/ 57646
18 placebo/ 422506
19 "control group"/ 110888
20 (random* or sham or placebo*).ti,ab,hw,kf. 2785191
21 ((singl* or doubl*) adj (blind* or dumm* or mask*)).ti,ab,hw,kf. 388248
22 ((tripl* or trebl*) adj (blind* or dumm* or mask*)).ti,ab,hw,kf. 2600
23 (control* adj3 (study or studies or trial* or group*)).ti,ab,kf. 1884395

24 (nonrandom* or non random* or non-random* or quasi-random* or quasirandom*).ti,ab,hw,kf. 77146
25 allocated.ti,ab,hw. 120076
26 ((open label or open-label) adj5 (study or studies or trial*)).ti,ab,hw,kf. 97369
27 ((equivalence or superiority or non-inferiority or noninferiority) adj3 (study or studies or trial*)).ti,ab,hw,kf. 21385
28 (pragmatic study or pragmatic studies).ti,ab,hw,kf. 1081
29 ((pragmatic or practical) adj3 trial*).ti,ab,hw,kf. 10262
30 ((quasiexperimental or quasi-experimental) adj3 (study or studies or trial*)).ti,ab,hw,kf. 23060
31 (phase adj3 (III or "3") adj3 (study or studies or trial*)).ti,hw,kf. 144092
32 or/11-31 4100354
33 10 and 32 5723
34 33 and 2020:2024.(yr). 2276
35 34 and English.lg. 2242
36 35 not (exp animal/ not exp human/) 2216
37 36 not (review.pt. or (exp "review"/ or "systematic review"/ or exp "meta analysis"/ or "systematic review (topic)"/ or "meta analysis (topic)"/ or ("rapid review" or "systematic review" or "scoping review" or "umbrella review" or "meta analy*" or "metanaly*" or "metaanaly*" or "met analy*" or "narrative review" or "literature review").ti.)) 1739
38 ("music therapy"/ and English.lg. and 2020:2024.(yr). and 32) not (review.pt. or (exp "review"/ or "systematic review"/ or exp "meta analysis"/ or "systematic review (topic)"/ or "meta analysis (topic)"/ or ("rapid review" or "systematic review" or "scoping review" or "umbrella review" or "meta analy*" or "metanaly*" or "metaanaly*" or "met analy*" or "narrative review" or "literature review").ti.)) 941
39 38 not 37 94
40 39 not (exp animal/ not exp human/) 84

CINAHL Complete (EBSCOhost) (Run on January 3, 2025, 721 records)

#	Query	Results
S1	(MH "Music Therapy")	7,210
S2	(MH "Music") AND (MW "Psychology")	284
S3	((MH "Music") OR (MH "Singing")) AND ((MH "Psychotherapy") OR (MH "Rehabilitation") OR (MH "Therapeutics") OR (MH "Guided Imagery") OR (MH "Sensory Stimulation") OR (MH "Acoustic Stimulation") OR (MW "Therapy" OR MW "Rehabilitation"))	1,048
S4	TI ((music* AND (imagery OR intervention* OR making OR medicine OR therap* OR treatment* OR rehabilitation))) OR SU ((music* AND (imagery OR intervention* OR making OR medicine OR therap* OR treatment* OR rehabilitation)))	9,564
S5	AB ((music* N3 (imagery OR intervention* OR making OR medicine OR therap* OR treatment* OR rehabilitation)))	3,922
S6	TI musicotherapy OR AB musicotherapy OR SU musicotherapy	7
S7	TI (((music* OR singing OR singer OR singers OR song OR songs OR vocal OR vocalization) AND (psychotherap* OR "sensory therap*" OR "acoustic stimulation"))	1,041

	) OR AB (((music* OR singing OR singer OR singers OR song OR songs OR vocal OR vocalization) AND (psychotherap* OR "sensory therap*" OR "acoustic stimulation"))) OR SU (((music* OR singing OR singer OR singers OR song OR songs OR vocal OR vocalization) AND (psychotherap* OR "sensory therap*" OR "acoustic stimulation")))	
S8	TI ((music* N3 (activit* OR composition OR instrument* OR improvisation OR listen* OR performance))) OR AB ((music* N3 (activit* OR composition OR instrument* OR improvisation OR listen* OR performance))) OR SU ((music* N3 (activit* OR composition OR instrument* OR improvisation OR listen* OR performance)))	2,904
S9	TI (((chant OR Chants OR songwriting OR song-writing OR "rhythmic auditory stimulation" OR melody OR melodies OR lullaby OR lullabies OR "Hip Hop" OR (Rock N2 Roll) OR (music N2 (ambient OR classic* OR ethnic OR folk OR jazz OR meditative OR metal OR pop OR popular OR religious OR rock OR sacred OR spiritual OR "symphon* traditional"))) AND (imagery OR intervention* OR therap* OR treatment* OR rehabilitation))) OR AB (((chant OR Chants OR songwriting OR song-writing OR "rhythmic auditory stimulation" OR melody OR melodies OR lullaby OR lullabies OR jazz OR "Hip Hop" OR (Rock N2 Roll) OR (music N2 (ambient OR classic* OR ethnic OR folk OR jazz OR meditative OR metal OR pop OR popular OR religious OR rock OR sacred OR spiritual OR "symphon* traditional"))) AND (imagery OR intervention* OR therap* OR treatment* OR rehabilitation))) OR SU (((chant OR Chants OR songwriting OR song-writing OR "rhythmic auditory stimulation" OR melody OR melodies OR lullaby OR lullabies OR jazz OR "Hip Hop" OR (Rock N2 Roll) OR (music N2 (ambient OR classic* OR ethnic OR folk OR jazz OR meditative OR metal OR pop OR popular OR religious OR rock OR sacred OR spiritual OR "symphon* traditional"))) AND (imagery OR intervention* OR therap* OR treatment* OR rehabilitation)))	621
S10	S1 OR S2 OR S3 OR S4 OR S5 OR S6 OR S7 OR S8 OR S9	12,497
S11	((PT "Randomized Controlled Trial") OR (PT "Controlled Clinical Trial") OR (PT "Pragmatic Clinical Trial") OR (PT "Equivalence Trial") OR (PT "Clinical Trial, Phase III"))	160,900
S12	(MH "Randomized Controlled Trials")	148,389
S13	(MH "Random Assignment")	89,284
S14	(MH "Double-Blind Studies")	54,612
S15	(MH "Single-Blind Studies")	16,224
S16	(MH "Placebos")	14,579
S17	(MH "Control Group")	15,348
S18	TI ((random* OR sham OR placebo*)) OR AB ((random* OR sham OR placebo*)) OR SU ((random* OR sham OR placebo*))	570,646

S19	TI (((singl* OR doubl*) W1 (blind* OR dumm* OR mask*))) OR AB (((singl* OR doubl*) W1 (blind* OR dumm* OR mask*))) OR SU (((singl* OR doubl*) W1 (blind* OR dumm* OR mask*)))	89,932
S20	TI (((tripl* OR trebl*) W1 (blind* OR dumm* OR mask*))) OR AB (((tripl* OR trebl*) W1 (blind* OR dumm* OR mask*))) OR SU (((tripl* OR trebl*) W1 (blind* OR dumm* OR mask*)))	887
S21	TI ((control* N3 (study OR studies OR trial* OR group*))) OR AB ((control* N3 (study OR studies OR trial* OR group*))) OR SU ((control* N3 (study OR studies OR trial* OR group*)))	513,718
S22	TI ((Nonrandom* OR "non random*" OR non-random* OR quasi-random* OR quasirandom*)) OR AB ((Nonrandom* OR "non random*" OR non-random* OR quasi-random* OR quasirandom*)) OR SU ((Nonrandom* OR "non random*" OR non-random* OR quasi-random* OR quasirandom*))	16,283
S23	(TI allocated OR AB allocated OR SU allocated)	26,116
S24	TI ((("open label" OR open-label) N5 (study OR studies OR trial*))) OR AB ((("open label" OR open-label) N5 (study OR studies OR trial*))) OR SU ((("open label" OR open-label) N5 (study OR studies OR trial*)))	17,032
S25	TI (((equivalence OR superiority OR non-inferiority OR noninferiority) N3 (study OR studies OR trial*))) OR AB (((equivalence OR superiority OR non-inferiority OR noninferiority) N3 (study OR studies OR trial*))) OR SU (((equivalence OR superiority OR non-inferiority OR noninferiority) N3 (study OR studies OR trial*)))	5,968
S26	TI (("pragmatic study" OR "pragmatic studies")) OR AB (("pragmatic study" OR "pragmatic studies")) OR SU (("pragmatic study" OR "pragmatic studies"))	289
S27	TI (((pragmatic OR practical) N3 trial*)) OR AB (((pragmatic OR practical) N3 trial*)) OR SU (((pragmatic OR practical) N3 trial*))	4,171
S28	TI (((quasiexperimental OR quasi-experimental) N3 (study OR studies OR trial*))) OR AB (((quasiexperimental OR quasi-experimental) N3 (study OR studies OR trial*))) OR SU (((quasiexperimental OR quasi-experimental) N3 (study OR studies OR trial*)))	22,974
S29	TI ((phase N3 (III OR 3) N3 (study OR studies OR trial*))) OR AB ((phase N3 (III OR 3) N3 (study OR studies OR trial*))) OR SU ((phase N3 (III OR 3) N3 (study OR studies OR trial*)))	20,217
S30	S11 OR S12 OR S13 OR S14 OR S15 OR S16 OR S17 OR S18 OR S19 OR S20 OR S21 OR S22 OR S23 OR S24 OR S25 OR S26 OR S27 OR S28 OR S29	822,365
S31	S10 AND S30	2,676
S32	S31 AND ((PY 2020) OR (PY 2021) OR (PY 2022) OR (PY 2023) OR (PY 2024))	949
S33	S32 AND (LA English)	896

S34	S33 NOT ((MH "Animals+") NOT (MH "Humans+"))	895
S35	S34 NOT ((MH "systematic review" OR MH "meta analysis" OR MH "Technology, Medical/EV" OR PT "systematic review" OR PT "meta analysis") OR ((TI "rapid review") OR (TI "systematic review") OR (TI "scoping review") OR (TI "umbrella review") OR (TI "meta analy*") OR (TI metanaly*) OR (TI metaanaly*) OR (TI "met analy*") OR (TI "narrative review") OR (TI "literature review")))	721

PsycINFO 1887-current (EBSCOhost) (Run on January 3, 2025, 652 records)

#	Query	Results
S1	DE "Music Therapy"	6,560
S2	(DE "Music") AND (DE "Psychology")	361
S3	((DE "Music") OR (DE "singing")) AND ((DE "psychotherapy") OR (DE "rehabilitation") OR (DE "Intervention") OR (DE "Guided Imagery") OR (DE "Sensory Integration") OR (DE "Auditory Stimulation") OR (DE "Therapy"))	2,239
S4	TI ((music* AND (imagery OR intervention* OR making OR medicine OR therap* OR treatment* OR rehabilitation))) OR SU ((music* AND (imagery OR intervention* OR making OR medicine OR therap* OR treatment* OR rehabilitation)))	9,342
S5	AB ((music* N3 (imagery OR intervention* OR making OR medicine OR therap* OR treatment* OR rehabilitation)))	8,407
S6	TI musicotherapy OR AB musicotherapy OR SU musicotherapy	30
S7	TI (((music* OR singing OR singer OR singers OR song OR songs OR vocal OR vocalization) AND (psychotherap* OR "sensory therap*" OR "acoustic stimulation"))) OR AB (((music* OR singing OR singer OR singers OR song OR songs OR vocal OR vocalization) AND (psychotherap* OR "sensory therap*" OR "acoustic stimulation"))) OR SU (((music* OR singing OR singer OR singers OR song OR songs OR vocal OR vocalization) AND (psychotherap* OR "sensory therap*" OR "acoustic stimulation")))	4,032
S8	TI ((music* N3 (activit* OR composition OR instrument* OR improvisation OR listen* OR performance))) OR AB ((music* N3 (activit* OR composition OR instrument* OR improvisation OR listen* OR performance))) OR SU ((music* N3 (activit* OR composition OR instrument* OR improvisation OR listen* OR performance)))	11,852
S9	TI (((chant OR Chants OR songwriting OR song-writing OR "rhythmic auditory stimulation" OR melody OR melodies OR lullaby OR lullabies OR "Hip Hop" OR (Rock N2 Roll) OR (music N2 (ambient OR classic* OR ethnic OR folk OR jazz OR meditative OR metal OR pop OR popular OR religious OR rock OR sacred OR spiritual OR "symphon* traditional"))) AND (imagery OR intervention* OR therap* OR treatment* OR rehabilitation))) OR AB (((chant OR Chants OR songwriting OR song-writing OR "rhythmic auditory stimulation" OR melody OR melodies OR lullaby OR lullabies OR jazz OR "Hip Hop" OR (Rock N2 Roll) OR (music N2 (ambient OR classic* OR ethnic OR folk OR jazz OR meditative OR metal OR pop OR popular OR religious OR rock OR	1,325

	sacred OR spiritual OR "symphon* traditional"))) AND (imagery OR intervention* OR therap* OR treatment* OR rehabilitation))) OR SU (((chant OR Chants OR songwriting OR song-writing OR "rhythmic auditory stimulation" OR melody OR melodies OR lullaby OR lullabies OR jazz OR "Hip Hop" OR (Rock N2 Roll) OR (music N2 (ambient OR classic* OR ethnic OR folk OR jazz OR meditative OR metal OR pop OR popular OR religious OR rock OR sacred OR spiritual OR "symphon* traditional"))) AND (imagery OR intervention* OR therap* OR treatment* OR rehabilitation)))	
S10	S1 OR S2 OR S3 OR S4 OR S5 OR S6 OR S7 OR S8 OR S9	23,872
S11	DE "Randomized Clinical Trials"	587
S12	DE "Randomized Controlled Trials"	1,126
S13	DE "Random Sampling"	1,183
S14	DE "Placebo"	6,828
S15	DE "Experiment Controls"	976
S16	TI ((random* OR sham OR placebo*)) OR AB ((random* OR sham OR placebo*)) OR SU ((random* OR sham OR placebo*))	306,710
S17	TI (((singl* OR doubl*) W1 (blind* OR dumm* OR mask*))) OR AB (((singl* OR doubl*) W1 (blind* OR dumm* OR mask*))) OR SU (((singl* OR doubl*) W1 (blind* OR dumm* OR mask*)))	38,459
S18	TI (((tripl* OR trebl*) W1 (blind* OR dumm* OR mask*))) OR AB (((tripl* OR trebl*) W1 (blind* OR dumm* OR mask*))) OR SU (((tripl* OR trebl*) W1 (blind* OR dumm* OR mask*)))	146
S19	TI ((control* N3 (study OR studies OR trial* OR group*))) OR AB ((control* N3 (study OR studies OR trial* OR group*))) OR SU ((control* N3 (study OR studies OR trial* OR group*)))	254,084
S20	TI ((Nonrandom* OR "non random*" OR non-random* OR quasi-random* OR quasirandom*)) OR AB ((Nonrandom* OR "non random*" OR non-random* OR quasi-random* OR quasirandom*)) OR SU ((Nonrandom* OR "non random*" OR non-random* OR quasi-random* OR quasirandom*))	7,181
S21	(TI allocated OR AB allocated OR SU allocated)	14,079
S22	TI ((("open label" OR open-label) N5 (study OR studies OR trial*))) OR AB ((("open label" OR open-label) N5 (study OR studies OR trial*))) OR SU ((("open label" OR open-label) N5 (study OR studies OR trial*)))	5,774
S23	TI (((equivalence OR superiority OR non-inferiority OR noninferiority) N3 (study OR studies OR trial*))) OR AB (((equivalence OR superiority OR non-inferiority OR noninferiority) N3 (study OR studies OR trial*))) OR SU (((equivalence OR superiority OR non-inferiority OR noninferiority) N3 (study OR studies OR trial*)))	1,748

S24	TI (("pragmatic study" OR "pragmatic studies")) OR AB (("pragmatic study" OR "pragmatic studies")) OR SU (("pragmatic study" OR "pragmatic studies"))	162
S25	TI (((pragmatic OR practical) N3 trial*)) OR AB (((pragmatic OR practical) N3 trial*)) OR SU (((pragmatic OR practical) N3 trial*))	1,534
S26	TI (((quasiexperimental OR quasi-experimental) N3 (study OR studies OR trial*))) OR AB (((quasiexperimental OR quasi-experimental) N3 (study OR studies OR trial*))) OR SU (((quasiexperimental OR quasi-experimental) N3 (study OR studies OR trial*)))	8,632
S27	TI ((phase N3 (III OR 3) N3 (study OR studies OR trial*))) OR AB ((phase N3 (III OR 3) N3 (study OR studies OR trial*))) OR SU ((phase N3 (III OR 3) N3 (study OR studies OR trial*)))	3,474
S28	S11 OR S12 OR S13 OR S14 OR S15 OR S16 OR S17 OR S18 OR S19 OR S20 OR S21 OR S22 OR S23 OR S24 OR S25 OR S26 OR S27	476,082
S29	S10 AND S28	2,934
S30	S29 AND ((PY 2020) OR (PY 2021) OR (PY 2022) OR (PY 2023) OR (PY 2024))	788
S31	S30 AND (LA English)	773
S32	S31 NOT ((DE "Animals" OR PO=Animal OR DE "Animal Research") NOT (DE "Human" OR PO=Human))	770
S33	S32 NOT ((PZ review*) OR (DE Literature Review) OR (DE Systematic Review) OR (DE Meta Analysis) OR (TI "rapid review") OR (TI "systematic review") OR (TI "scoping review") OR (TI "umbrella review") OR (TI "meta analy*") OR (TI metanaly*) OR (TI metaanaly*) OR (TI "met analy*") OR (TI "narrative review") OR (TI "literature review"))	652

Supplemental File 3
Search 2: Music perception-music neuroscience studies
Search strategy

MEDLINE(R) ALL (Ovid) (Run on January 3, 2025, 1328 records)

1 ("Acoustic Stimulation"/ or "Auditory Perception"/ or "Pitch Perception"/) and Music/ 4919
2 (music* adj3 (detection or perception or cognition or processing or recognition)).ti,ab,kf. 2783
3 (tonal adj3 processing).ti,ab,kf. 77
4 (music* and ((harmonic or melodic or pitch or rhythm or temporal) adj3 perception)).ti,ab,kf. 732
5 or/1-4 6639
6 exp Neurosciences/ 25511
7 Brain/ 575378
8 exp "Neural Pathways"/ 119439
9 exp Cognition/ 213883
10 exp "Psychomotor Performance"/ 123874
11 "Brain Mapping"/ 101802
12 Functional Neuroimaging/ 4840
13 "magnetic resonance imaging"/ 501794
14 Echoencephalography/ 3885
15 Electroencephalography/ 168253
16 Magnetoencephalography/ 9742
17 exp "Positron-Emission Tomography"/ 85685
18 "Brain Waves"/ 4753
19 (neuro* or neural or neuroscience or neurobiolog* or neurocogniti* or psychophysiolog* or psycholog* or (cognition or cognitive)).ti,ab,kf. 3290345
20 ("magnetic resonance imaging" or (fMRI or MRI) or neuroimaging or (EEG or MEG or MEEG) or encephalogra* or electroencephalogra* or Echoencephalo* or magnetoencephalogr* or ("Brain PET" or "PET scan*" or "PET Imaging") or "positron emission tomography").ti,ab,kf. 801785
21 ("Audiomotor Processing" or kinemat* or "motor control" or "rhythm processing" or "predictive processing").ti,ab,kf. 68496
22 or/6-21 4449474
23 Music/ and (Affect/ or Arousal/ or Emotions/ or Pleasure/ or Reward/) 1821
24 (music* and (reward or valence or "peak experience" or arousal or "emotional contagion" or "affective response*" or "cognitive response*" or "audio-visual perception" or "emotional response*" or pleasure or "pleasurable")).ti,ab,kf. 1602
25 or/23-24 2825
26 (5 and 22) or 25 6139
27 (experiment* or study or cross-over or investigat* or random* or condition* or subjects or participants or rating* or self-report* or measur* or scale*).ti,ab,kf. 18212896
28 26 and 27 4904
29 28 and 2020:2024.(yr). 1465
30 29 and English.lg. 1458
31 30 not (exp animals/ not exp humans/) 1437
32 31 not ((review or "systematic review" or "meta-analysis").pt. or ("review literature as topic"/ or "systematic reviews as topic"/ or "Meta-Analysis as Topic"/) or ("rapid review" or "systematic review" or

"scoping review" or "umbrella review" or "meta analy*" or "metanaly*" or "metaanaly*" or "met analy*" or "narrative review" or "literature review").ti.) 1328

Embase (Ovid) (Run on January 3, 2025, 1858 records)

1 ("auditory stimulation"/ or hearing/ or "pitch perception"/) and music/ 4395
2 (music* adj3 (detection or perception or cognition or processing or recognition)).ti,ab,kf. 3000
3 (tonal adj3 processing).ti,ab,kf. 89
4 (music* and ((harmonic or melodic or pitch or rhythm or temporal) adj3 perception)).ti,ab,kf. 776
5 or/1-4 6571
6 exp neuroscience/ 273857
7 brain/ 366433
8 exp "nerve tract"/ 69996
9 exp cognition/ 3246583
10 exp "psychomotor performance"/ 219709
11 "brain mapping"/ 38348
12 "functional neuroimaging"/ 18059
13 "nuclear magnetic resonance imaging"/ 1101930
14 echoencephalography/ 3821
15 electroencephalography/ 133104
16 magnetoencephalography/ 15652
17 exp "positron emission tomography"/ 262023
18 electroencephalogram/ 145223
19 (neuro* or neural or neuroscience or neurobiolog* or neurocogniti* or psychophysiolog* or psycholog* or (cognition or cognitive)).ti,ab,kf. 4308200
20 ("magnetic resonance imaging" or (fMRI or MRI) or neuroimaging or (EEG or MEG or MEEG) or encephalogra* or electroencephalogra* or Echoencephalo* or magnetoencephalogr* or ("Brain PET" or "PET scan*" or "PET Imaging") or "positron emission tomography").ti,ab,kf. 1202592
21 ("Audiomotor Processing" or kinemat* or "motor control" or "rhythm processing" or "predictive processing").ti,ab,kf. 81272
22 or/6-21 7878225
23 music/ and (affect/ or arousal/ or emotion/ or pleasure/ or reward/) 2827
24 (music* and (reward or valence or "peak experience" or arousal or "emotional contagion" or "affective response*" or "cognitive response*" or "audio-visual perception" or "emotional response*" or pleasure or pleasurable)).ti,ab,kf. 1809
25 or/23-24 3621
26 (5 and 22) or 25 8390
27 (experiment* or study or cross-over or investigat* or random* or condition* or subjects or participants or rating* or self-report* or measur* or scale*).ti,ab,kf. 22822873
28 26 and 27 6662
29 28 and 2020:2024.(yr). 2033
30 29 and English.lg. 2021
31 30 not (exp animals/ not exp humans/) 1991
32 31 not (review.pt. or (exp "review"/ or "systematic review"/ or exp "meta analysis"/ or "systematic review (topic)"/ or "meta analysis (topic)"/) or ("rapid review" or "systematic review"

or "scoping review" or "umbrella review" or "meta analy*" or "metanaly*" or "metaanaly*" or "met analy*" or "narrative review" or "literature review").ti.) 1858

CINAHL Complete (EBSCOhost) (Run on January 3, 2025, 296 records)

#	Query	Results
S1	((MH "Acoustic Stimulation") OR (MH "Auditory Perception") OR (MH "Pitch Perception")) AND (MH "Music")	896
S2	TI ((music* N3 (detection OR perception OR cognition OR processing OR recognition))) OR AB ((music* N3 (detection OR perception OR cognition OR processing OR recognition))) OR SU ((music* N3 (detection OR perception OR cognition OR processing OR recognition)))	714
S3	TI (tonal N3 processing) OR AB (tonal N3 processing) OR SU (tonal N3 processing)	15
S4	TI ((music* AND ((harmonic OR melodic OR pitch OR rhythm OR temporal) N3 perception))) OR AB ((music* AND ((harmonic OR melodic OR pitch OR rhythm OR temporal) N3 perception))) OR SU ((music* AND ((harmonic OR melodic OR pitch OR rhythm OR temporal) N3 perception)))	344
S5	S1 OR S2 OR S3 OR S4	1,372
S6	(MH "Neurosciences+")	7,702
S7	(MH "Brain")	64,209
S8	(MH "Neural Pathways+")	7,119
S9	(MH "Cognition+")	88,041
S10	(MH "Psychomotor Performance+")	46,065
S11	(MH "Brain Mapping")	8,880
S12	(MH "Neuroradiography")	7,998
S13	(MH "Magnetic Resonance Imaging")	146,201
S14	(MH "Echoencephalography")	403
S15	(MH "Electroencephalography")	19,521
S16	(MH "Electromagnetic Fields")	2,171
S17	(MH "Positron-Emission Tomography+")	2,071
S18	(MH "Brain Waves")	193
S19	TI ((neuro* OR neural OR neuroscience OR neurobiolog* OR neurocogniti* OR psychophysiolog* OR psycholog* OR (cognition OR cognitive))) OR AB ((neuro* OR	941,526

	neural OR neuroscience OR neurobiolog* OR neurocogniti* OR psychophysiolog* OR psycholog* OR (cognition OR cognitive))) OR SU ((neuro* OR neural OR neuroscience OR neurobiolog* OR neurocogniti* OR psychophysiolog* OR psycholog* OR (cognition OR cognitive)))	
S20	TI (("magnetic resonance imaging" OR (fMRI OR MRI) OR neuroimaging OR (EEG OR MEG OR MEEG) OR encephalogra* OR electroencephalogra* OR Echoencephalo* OR magnetoencephalogr* OR ("Brain PET" OR "PET scan*" OR "PET Imaging") OR "positron emission tomography")) OR AB (("magnetic resonance imaging" OR (fMRI OR MRI) OR neuroimaging OR (EEG OR MEG OR MEEG) OR encephalogra* OR electroencephalogra* OR Echoencephalo* OR magnetoencephalogr* OR ("Brain PET" OR "PET scan*" OR "PET Imaging") OR "positron emission tomography")) OR SU (("magnetic resonance imaging" OR (fMRI OR MRI) OR neuroimaging OR (EEG OR MEG OR MEEG) OR encephalogra* OR electroencephalogra* OR Echoencephalo* OR magnetoencephalogr* OR ("Brain PET" OR "PET scan*" OR "PET Imaging") OR "positron emission tomography"))	229,889
S21	TI (("Audiomotor Processing" OR kinemat* OR "motor control" OR "rhythm processing" OR "predictive processing")) OR AB (("Audiomotor Processing" OR kinemat* OR "motor control" OR "rhythm processing" OR "predictive processing")) OR SU (("Audiomotor Processing" OR kinemat* OR "motor control" OR "rhythm processing" OR "predictive processing"))	29,999
S22	S6 OR S7 OR S8 OR S9 OR S10 OR S11 OR S12 OR S13 OR S14 OR S15 OR S16 OR S17 OR S18 OR S19 OR S20 OR S21	1,169,721
S23	(MH Music) AND ((MH Affect) OR (MH Arousal) OR (MH Emotions) OR (MH Pleasure) OR (MH Reward))	733
S24	TI ((music* AND (reward OR valence OR "peak experience" OR arousal OR "emotional contagion" OR "affective response*" OR "cognitive response*" or "audio-visual perception" OR "emotional response*" OR pleasure OR pleasurable))) OR AB ((music* AND (reward OR valence OR "peak experience" OR arousal OR "emotional contagion" OR "affective response*" OR "cognitive response*" or "audio-visual perception" OR "emotional response*" OR pleasure OR pleasurable))) OR SU ((music* AND (reward OR valence OR "peak experience" OR arousal OR "emotional contagion" OR "affective response*" OR "cognitive response*" or "audio-visual perception" OR "emotional response*" OR pleasure OR pleasurable))	469
S25	S23 OR S24	1,016
S26	(S5 AND S22) OR S25	1,542
S27	TI ((experiment* OR study OR cross-over OR investigat* OR random* OR condition* OR subjects OR participants OR rating* OR self-report* OR measur* OR scale*)) OR AB ((experiment* OR study OR cross-over OR investigat* OR random* OR condition* OR subjects OR participants OR rating* OR self-report* OR measur* OR scale*)) OR SU ((experiment* OR study OR cross-over OR investigat* OR random* OR condition* OR subjects OR participants OR rating* OR self-report* OR measur* OR scale*))	3,941,900

S28	S26 AND S27	1,145
S29	S28 AND ((PY 2020) OR (PY 2021) OR (PY 2022) OR (PY 2023) OR (PY 2024))	327
S30	S29 AND (LA English)	319
S31	S30 NOT ((MH "Animals+") NOT (MH "Humans+"))	318
S32	S31 NOT ((MH "systematic review" OR MH "meta analysis" OR MH "literature review" OR PT "systematic review" OR PT "meta analysis") OR ((TI "rapid review") OR (TI "systematic review") OR (TI "scoping review") OR (TI "umbrella review") OR (TI "meta analy*") OR (TI metanaly*) OR (TI metaanaly*) OR (TI "met analy*") OR (TI "narrative review") OR (TI "literature review")))	296

PsycINFO 1887-current (EBSCOhost) (Run on January 3, 2025, 1239 records)

#	Query	Results
S1	((DE "Acoustic Stimulation") OR (DE "Auditory Perception") OR (DE "Pitch Perception")) AND (DE Music)	4,047
S2	TI ((music* N3 (detection OR perception OR cognition OR processing OR recognition))) OR AB ((music* N3 (detection OR perception OR cognition OR processing OR recognition))) OR SU ((music* N3 (detection OR perception OR cognition OR processing OR recognition)))	5,980
S3	TI (tonal N3 processing) OR AB (tonal N3 processing) OR SU (tonal N3 processing)	100
S4	TI ((music* AND ((harmonic OR melodic OR pitch OR rhythm OR temporal) N3 perception))) OR AB ((music* AND ((harmonic OR melodic OR pitch OR rhythm OR temporal) N3 perception))) OR SU ((music* AND ((harmonic OR melodic OR pitch OR rhythm OR temporal) N3 perception)))	1,654
S5	S1 OR S2 OR S3 OR S4	8,928
S6	DE "Neurosciences"	19,309
S7	DE "Brain"	138,614
S8	DE "Neural Pathways"	24,292
S9	DE "Cognition"	88,632
S10	DE "Perceptual Motor Processes"	15,585
S11	DE "Stereotaxic Atlas"	4,975
S12	DE "Neuroimaging"	23,276
S13	DE "Magnetic Resonance Imaging"	71,840
S14	DE "Echoencephalography"	152
S15	DE "Electroencephalography"	60,976

S16	DE "Magnetoencephalography"	5,440
S17	DE "Positron Emission Tomography"	4,923
S18	DE "Brain Waves"	1,481
S19	TI ((neuro* OR neural OR neuroscience OR neurobiolog* OR neurocogniti* OR psychophysiolog* OR psycholog* OR (cognition OR cognitive))) OR AB ((neuro* OR neural OR neuroscience OR neurobiolog* OR neurocogniti* OR psychophysiolog* OR psycholog* OR (cognition OR cognitive))) OR SU ((neuro* OR neural OR neuroscience OR neurobiolog* OR neurocogniti* OR psychophysiolog* OR psycholog* OR (cognition OR cognitive)))	2,150,566
S20	TI (("magnetic resonance imaging" OR (fMRI OR MRI) OR neuroimaging OR (EEG OR MEG OR MEEG) OR encephalogra* OR electroencephalogra* OR Echoencephalo* OR magnetoencephalogr* OR ("Brain PET" OR "PET scan*" OR "PET Imaging") OR "positron emission tomography")) OR AB (("magnetic resonance imaging" OR (fMRI OR MRI) OR neuroimaging OR (EEG OR MEG OR MEEG) OR encephalogra* OR electroencephalogra* OR Echoencephalo* OR magnetoencephalogr* OR ("Brain PET" OR "PET scan*" OR "PET Imaging") OR "positron emission tomography")) OR SU (("magnetic resonance imaging" OR (fMRI OR MRI) OR neuroimaging OR (EEG OR MEG OR MEEG) OR encephalogra* OR electroencephalogra* OR Echoencephalo* OR magnetoencephalogr* OR ("Brain PET" OR "PET scan*" OR "PET Imaging") OR "positron emission tomography"))	221,216
S21	TI (("Audiomotor Processing" OR kinemat* OR "motor control" OR "rhythm processing" OR "predictive processing")) OR AB (("Audiomotor Processing" OR kinemat* OR "motor control" OR "rhythm processing" OR "predictive processing")) OR SU (("Audiomotor Processing" OR kinemat* OR "motor control" OR "rhythm processing" OR "predictive processing"))	17,110
S22	S6 OR S7 OR S8 OR S9 OR S10 OR S11 OR S12 OR S13 OR S14 OR S15 OR S16 OR S17 OR S18 OR S19 OR S20 OR S21	2,230,316
S23	(DE Music) AND ((DE Affect) OR (DE Arousal) OR (DE Emotions) OR (DE Pleasure) OR (DE Reward))	1,955
S24	TI ((music* AND (reward OR valence OR "peak experience" OR arousal OR "emotional contagion" OR "affective response*" OR "cognitive response*" or "audio-visual perception" OR "emotional response*" OR pleasure OR pleasurable))) OR AB ((music* AND (reward OR valence OR "peak experience" OR arousal OR "emotional contagion" OR "affective response*" OR "cognitive response* or "audio-visual perception" OR "emotional response*" OR pleasure OR pleasurable))) OR SU ((music* AND (reward OR valence OR "peak experience" OR arousal OR "emotional contagion" OR "affective response*" OR "cognitive response*" or "audio-visual perception" OR "emotional response*" OR pleasure OR pleasurable)))	2,295
S25	S23 OR S24	3,684
S26	(S5 AND S22) OR S25	7,975

S27	TI ((experiment* OR study OR cross-over OR investigat* OR random* OR condition* OR subjects OR participants OR rating* OR self-report* OR measur* OR scale*)) OR AB ((experiment* OR study OR cross-over OR investigat* OR random* OR condition* OR subjects OR participants OR rating* OR self-report* OR measur* OR scale*)) OR SU ((experiment* OR study OR cross-over OR investigat* OR random* OR condition* OR subjects OR participants OR rating* OR self-report* OR measur* OR scale*))	3,992,653
S28	S26 AND S27	6,470
S29	S28 AND ((PY 2020) OR (PY 2021) OR (PY 2022) OR (PY 2023) OR (PY 2024))	1,311
S30	S29 AND (LA English)	1,279
S31	S30 NOT ((DE "Animals" OR PO=Animal OR DE "Animal Research") NOT (DE "Human" OR PO=Human))	1,277
S32	S31 NOT ((PZ review*) OR (DE Literature Review) OR (DE Systematic Review) OR (DE Meta Analysis) OR (TI "rapid review") OR (TI "systematic review") OR (TI "scoping review") OR (TI "umbrella review") OR (TI "meta analy*") OR (TI metanaly*) OR (TI metaanaly*) OR (TI "met analy*") OR (TI "narrative review") OR (TI "literature review"))	1,239

Supplemental File 4

Covidence Extraction Template

General

1. Should this article be flagged due to limited information about the music stimulus/intervention/experience?
    a. Yes (comment below)
    b. No
2. Country in which the study conducted
    a. United States
    b. UK
    c. Canada
    d. Australia
    e. Unclear/not reported
    f. Other

Methods

1. Aim of study:
2. Study Design
    a. Parallel
    b. Crossover/Repeated Measures
    c. Other
3. Randomization
    d. Randomized
    e. Nonrandomized

Participants

1. Population Type:
    a. Healthy Volunteers
    b. Clinical
2. If clinical, diagnosis or broad descriptors
3. Is the music experience/intervention/stimuli single or multicomponent?
    a. Single Component
    b. Multicomponent

COMPONENT 1: MUSIC FEATURES

1. Did the paper address *Compositional Features*?

a. Yes

b. No

2. Were the framework definitions for “*compositional features*” adequate?

a. Yes

b. No (comment below)

c. Not applicable (feature not addressed)

3. Did the authors report any *compositional features* that were not included in the framework?

a. Yes (comment below)

b. No

4. Did the paper address *Psychoacoustic Features*?

a. Yes

b. No

5. Were the framework definitions for “*psychoacoustic features*” adequate?

a. Yes

b. No (comment below)

c. Not applicable (features not addressed)

6. Did the authors report any *psychoacoustic features* that were not included in the framework?

a. Yes (comment below)

b. No

7. Did the paper address the *sociocultural features*?

a. Yes

b. No

8. Was the framework definition of “*sociocultural features*” adequate?

a. Yes

b. No (comment below)

c. not applicable (features not addressed)

6. Did the authors report any *sociocultural features* that were not included in the framework?

a. Yes (comment below)

b. No

COMPONENT 2: PERSON-SPECIFIC MUSIC FACTORS

1. Did the paper address factors that may be unique to an individual?

a. Yes

b. No

2. Did the paper address *Music Preference*?

a. Yes

b. No

3. Was the framework definition of “music preference” adequate?

a. Yes

b. No (comment below)

c. Not applicable (feature not addressed)

4. Did the paper address *Familiarity with Music*?

a. Yes

b. No

5. Was the framework definition of “*familiarity with music*” adequate?

a. Yes

b. No (comment below)

c. No applicable (feature not addressed)

6. Did the paper address *Prior Musical Training/Experience*?

a. Yes

b. No

7. Was the framework definition of “*musical training/experience*” adequate?

a. Yes

b. No (comment below)

c. No applicable (feature note addressed)

8. Did the paper address *Music-Evoked Responses*?

a. Yes

b. No

9. Was the framework definition for “*music-evoked responses*” adequate?

a. Yes

b. No

c. Not applicable (feature not addressed)

10. Did the paper address *Auditory Processing Related Factors*?

a. Yes

b. No

11. Was the framework definition for “*auditory processing related factors*” adequate?

a. Yes

b. No

c. Not applicable (feature not addressed)

12. Did the authors reporting any <u>person-specific music factors</u> that were not included in the framework?

a. Yes (comment below)

b. No

COMPONENT 3: ENGAGEMENT MODE

1. Did the paper address how a person engages in the music or music-based intervention?

a. Yes

b. No

2. Did the paper address *Listening to Music*?

a. Yes

b. No

3. Was the framework definition for "*listening to music*" adequate?

a. Yes

b. No

c. Not applicable (feature not addressed)

4. Did the paper address *Composing Music*?

a. Yes

b. No

5. Was the framework definition for "*composing music*" adequate?

a. Yes

b. No

c. Not applicable (feature not addressed)

6. Did the paper address *Recreating Music*?

a. Yes

b. No

7. Was the framework definition for "*recreating music*" adequate?

a. Yes

b. No

c. Not applicable (feature not addressed)

8. Did the paper address *Improvising Music*?

a. Yes

b. No

9. Was the framework definition for "*improvising*" adequate?

a. Yes

b. No

c. Not applicable (feature not addressed)

10. Did the paper address *Music Instruction*?

a. Yes

b. No

11. Was the framework definition for "*music instruction*" adequate?

a. Yes

b. No

c. Not applicable (feature not addressed)

12. Did the authors reporting any engagement modes that were not included in the framework?

a. Yes (comment below)

b. No

COMPONENT 4: MUSIC DELIVERY FEATURES

1. Did the paper address factors related to music delivery?

a. Yes

b. No

2. Did the paper address *the Delivery Schedule*?

a. Yes

b. No

3. Was the framework definition for "*delivery schedule*" adequate?

a. Yes

b. No

c. Not applicable (feature not addressed)

4. Did the paper address *the Delivery Method*?

a. Yes

b. No

5. Was the framework definition for “*delivery method*” adequate?

a. Yes

b. No

c. Not applicable (feature not addressed)

6. Did the paper address *Facilitator Qualifications*?

a. Yes

b. No

7. Was the framework definition for “*facilitator qualifications*” adequate?

a. Yes

b. No

c. Not applicable (feature not addressed)

8. Did the authors reporting any <u>music delivery factors</u> that were not included in the framework?

a. Yes (comment below)

b. No

COMPONENT 5: SOCIAL AND ENVIRONMENTAL CONTEXTS

1. Did the paper address factors related to social and environmental contexts?

a. Yes

b. No

2. Did the paper address *the Relational Context*?

a. Yes

b. No

3. Was the framework definition for “*relational context*” adequate?

a. Yes

b. No

c. Not applicable (feature not addressed)

4. Did the paper address *the Environmental Context*?

a. Yes

b. No

5. Was the framework definition for "*environmental context*" adequate?

a. Yes

b. No

c. Not applicable (feature not addressed)

8. Did the authors reporting any <u>social or environmental contexts</u> that were not included in the framework?

a. Yes (comment below)

b. No

COMPONENT 6: MUSIC SELECTION

1. Did the paper address by whom and how the music was selected for the music experience?

a. Yes

b. No

2. Did the paper address *the Person Selecting the Music*?

a. Yes

b. No

3. Was the framework definition for "*person selecting the music*" adequate?

a. Yes

b. No

c. Not applicable (feature not addressed)

4. Did the paper address *the Process for Selecting the Music*?

a. Yes

b. No

5. Was the framework definition for "*process for selecting the music*" adequate?

a. Yes

b. No

c. Not applicable (feature not addressed)

6. Did the authors reporting any ways of selecting music that were not included in the framework?

a. Yes (comment below)

b. No

COMPONENT 7: ADDITIONAL COMPONENTS

1. Did the paper address any other experiences supported by or used in combinations with music experiences?

a. Yes

b. No

2. Did the paper address *Body Movement*?

a. Yes

b. No

3. Was the framework definition for "*body movement*" adequate?

a. Yes

b. No

c. Not applicable (feature not addressed)

4. Did the paper address *Imagery*?

a. Yes

b. No

5. Was the framework definition for "*imagery*" adequate?

a. Yes

b. No

c. Not applicable (feature not addressed)

6. Did the paper address *Relaxation*?

a. Yes

b. No

7. Was the framework definition for "*relaxation*" adequate?

a. Yes

b. No

c. Not applicable (feature not addressed)

8. Did the paper address *Other Arts Media*?

a. Yes

b. No

9. Was the framework definition for "*other arts media*" adequate?

a. Yes

b. No

c. Not applicable (feature not addressed)

10. Did the paper address *Spiritual Practice*?

a. Yes

b. No

11. Was the framework definition for "*spiritual practice*" adequate?

a. Yes

b. No

c. Not applicable (feature not addressed)

12. Did the paper address *Analysis/Discussion*?

a. Yes

b. No

13. Was the framework definition for "*analysis/discussion*" adequate?

a. Yes

b. No

c. Not applicable (feature not addressed)

14. Did the paper address *Biofeedback*?

a. Yes

b. No

15. Was the framework definition for "*biofeedback*" adequate?

a. Yes

b. No

c. Not applicable (feature not addressed)

16. Did the paper address *Human Touch*?

a. Yes

b. No

17. Was the framework definition for "*human touch*" adequate?

a. Yes

b. No

c. Not applicable (feature not addressed)

18. Did the authors report any *additional components* that were not included in the framework?

a. Yes (comment below)

b. No

DID THE AUTHORS IDENTIFY OTHER MUSIC OR MUSIC-BASED INTERVENTION COMPONENTS NOT CAPTURED IN THE CURRENT FRAMEWORK?

a. Yes (comment below)

b. No

Supplemental File 5

MBI Taxonomy

1. Music Features: Components, Subcomponents, and Definitions

**Musical Features**		**Refers to the compositional, psychoacoustic, and sociocultural elements of music.**
Compositional Features		Refers to the five elements of music: melody, harmony, rhythm, color (texture/sound) and form as well as genre and tonality.
**Component**	Subcomponent	Definition
**Melody**		A consecutive series of sounds (pitches) and silences (rests) arranged in time (rhythm).[1]
**Harmony**		Two or more pitches sounding simultaneously to produce chords that may be perceived as either stable (consonant) or unstable (dissonant). Successive chords a produce a harmonic progression.[1]
	Consonant Harmony	A chord (simultaneous sounding of two or more pitches) that is heard as being stable, pleasing, or complete within the tonal/modal norms of a particular culture.[1]
	Dissonant Harmony	A chord that is heard as being unstable, tense, transitional, or in need of resolution within the tonal/modal norms of a particular culture.[1]
**Rhythm**		The pattern of musical elements in time, including tempo, meter, syncopation, and silence (rests).[1,2]
	Meter	Pattern of strong and weak beats (or pulses) which organize a piece of music.[1]
	Syncopation	Compositional device that destabilizes the expected or established rhythmic pattern (meter) and creates a sense of anticipation for the accented beat (e.g., through displacing accent on weak beat, resting on strong beat).
	Tempo	The speed at which a piece of music is performed (often reported as beats-per-minute).[1]
**Sound**		Aspects of sound include timbre, texture, dynamics, and range/tessitura. [1]
	Timbre (color)	The unique sound produced by a particular instrument or voice or combination of instruments/voices.
	Texture	The overall quality or complexity of a piece of music, based on how each music feature manifests and relates to other features. Considerations include but are not limited to: presence/absence of accompaniment and number of melodic lines (e.g., monophony vs polyphony), presence/absence of lyrics, and vertical spacing of chords (i.e., density).

	Dynamics	Perceived variations in loudness and softness of music and how much contrast there is between the loudest and the quietest sounds. This is not the same thing as decibel volume or acoustic loudness (see Table 1. Psychoacoustic Features).
	Range/Tessitura	
	Range	Distance between the highest and lowest pitches.
	Tessitura	The portion of the full range most used or emphasized by an instrument or vocalist in a piece of music.
**Silence**		The intentional or unintentional absence of music or sound.
**Form**		The application and organization of all musical elements in a piece to create a sense of direction and shape through the use of repetition, contrast, and development. [1,2]
**Genre (compositional)**		Constructed categories of music that share a distinct set of musical characteristics or features such as a particular rhythmic pattern or instrumentation. Genres may also be constructed based on sociocultural, historical, or artistic contexts (see Table 1. Sociocultural Features).[3]
**Tonality (compositional)**		The relative organization of all the tones and harmonies of a piece of music and the relationships between them, often described in relation to a prominent, recurring note (tonic).[4]
**Psychoacoustic Features**		Refers to the physical parameters of sound including pitch, loudness, sharpness, tonality, duration, and roughness.
**Component**	Subcomponent	
**Pitch**		Subjective perception of a sound's position within the musical scale (i.e., high or low), determined by the objective frequency of the sound (i.e., rate of oscillation per second) (reported in hertz).[5]
**Loudness (amplitude)**		Subjective impression of the intensity of a sound (may be reported in decibels). [5,6]
**Sharpness**		Subjective perception of sounds with an emphasis on high frequencies as being harsh or piercing (e.g., cymbal crash).
**Booming**		Subjective perception of sounds with an emphasis on low frequencies as being resonant and impactful (e.g., bass drum).
**Tonality**		Indicator of tones that the ear perceives and draws out of a mass of sound.[7]
**Duration**		the length of time of a note or full piece of music is played.
**Roughness**		Subjective perception of sounds with an emphasis on rapid pitch fluctuations as being grating, unpleasant, or annoying (e.g., a

		poorly tuned string instrument or a wide vocal vibrato). [8]
**Salience**		The extent to which a sound stands out from other sound in the acoustic environment. [9]
**Sociocultural Features**		Refers to aspects of music (including genre) that reflect cultural identity,<br>societal values, and social functions, including but not limited to musical traditions/heritage, rituals, ceremonies, celebrations, healing practices, and political protext/activism.

2. Person-Specific Music Factors Components, Subcomponents, and Definitions

**Person-Specific Music Factors**		Refers to factors that may be unique to an individual. Person-specific factors are influenced by a range of dimensions including but not limited to life events, emotion, cognition, personality, self-identity, beliefs, preferences, and behaviors.
**Component**	Subcomponent	**Definition**
**Music Preference**		A person's liking for one piece of music, music genre, artist, or style as compared to another at a given point in time.[10-12]
**Familiarity with Music**		Feeling of being acquainted with a piece of music due to previous exposure to the piece or similar pieces. [13-15]
**Prior Musical Training/Experience**		Previous formal/informal music training or sustained engagement in vocal or instrumental music making.[16]
**Music-evoked Responses**		Refers to autobiographical, emotional, physical, and physiological responses evoked by music.
	Autobiographical memories/meaning	Memories and/or meaning evoked when hearing a piece of music from one's past.[17]
	Musical Valence and Arousal	Refers to the propensity of music to evoke a range of affective and emotional responses which may be associated with varying levels of physiological activation (i.e., arousal). [18]
	Spontaneous Movement	Unprompted movement that may occur when listening to or experiencing music.
	Auditory Processing Related Factors	Refers to conditions impacting the central nervous or auditory systems which affect auditory stimulus discrimination, auditory stimulus tolerance and/or how the brain processes sound information (e.g. autism, acquired brain injury, or hearing impairment).

3. Engagement Modes: Components and Definitions

**Engagement Mode**	Refers to how a person engages with music.
**Component**	**Definition**
**Listening to Music**	Experiencing live or recorded music. [19]
**Composing Music**	Composing Music. Writing songs, lyrics, or instrumental pieces or creating any kind of musical product. [20]
**Re-Creating Music**	Rendering, reproducing, realizing, or interpreting all or any part of an existing musical work. [20]<br>Re-creating can be instrumental, vocal, or computer generated.
**Improvising Music**	Extemporaneously creating music using instruments, voices, or other sound sources (e.g., body percussion; computer-generated sounds).
**Music Instruction**	Developing musical skills, knowledge, and understanding through a variety of methods, techniques, and approaches. [21]
**Audio- or Vibro- tactile Stimulation**	Providing tactile sensations that complement or enhance the auditory music listening experience. Often achieved through devices that convert musical sound into vibrations that can be felt on the skin. [22]

4. Music Delivery Factors: Components and Definitions

**Music Delivery Factors**	Refers to the music delivery schedule, delivery method, and facilitator qualifications.
**Component**	**Definition**
Delivery Schedule	Information about participants' exposure to a music experience (or music stimulus). This might include: (1) number of sessions (exposures); (2) length of each session (exposure) (e.g., minutes/h); (3) frequency of sessions – how often the exposure happens over a specified period of time (e.g., 3x/week); (4) time interval between sessions – when applicable, indicate time between sessions (exposures); and (5) duration of the program (or music exposures) over time (e.g., over 4 weeks). Delivery schedule might also include information about practice, experiences, or tasks that are assigned to participants between intervention or study sessions. [23]
Delivery Methods	Refers to the ways in which music is provided to or created with participants, (e.g., via live, recorded, or computer-generated means; via headphones or free field), including, if relevant, whether and how the music is adapted in the moment to intentionally influence participants' physiological or psychological responses (e.g., entrainment, iso principle).
Facilitator Qualifications	Refers to the professional background, credentials, training, and/or experience of the facilitator(s) of MBIs.

5. Social and Environmental Contexts: Components, Subcomponents, and Definitions

**Social and Environmental Contexts**		Refers to social, relational, and environmental circumstances under which a music experience (or stimulus) occurs, including the number of people involved, their role(s), and the surroundings. Music can be experienced or created alone, with another person, or in larger groups. [24]
**Component**	Subcomponent	Definition
**Relational Context**		Refers to the number of people involved in a music experience and whether the experience is facilitated.
	Number of People	Refers to the number of people involved in the music experience (alone, with another person, or in a group).
	Facilitate or Not Facilitated	Refers to whether a music-based experience was guided by a facilitator.
**Environmental Context**		Refers to the surroundings and conditions in which a music experience (or stimulus) occurs, such as location (e.g., home, lab, public space, virtual), presence of people other than participants/facilitators, or the presence of ambient sound.

6. Music Selection: Components and Definitions

**Music Selection**	Refers to the person(s) who select the music in a music research study and the process for making selections. [25]
**Component**	**Definition**
**Person(s) Selecting the Music**	The individuals who select music fall into three general categories: (1) the investigator(s), (2) the therapist(s) or facilitator(s), and (3) the participant(s), their caregiver(s), or family member(s). Selection may involve individuals from one or more than one of these categories.
**Process for Selecting the Music**	Process for Selecting the Music – The processes for selecting music used in a music research study fall into four general categories: (1) pre-identified parameters (e.g., specific music element such as tempo, algorithms or AI generation), (2) participant preference, (3) assessment, and/or (4) collaborative decision making.[26-29]

7. Additional Non-Music Components and Definitions

**Additional Non-Music Components**	Refers to components supported by or used in combination with music.[30-32]
**Component**	**Definition**
**Body Movement**	Prompted change(s) in the positioning of a body part(s) relative to the entire body. This can include (but is not limited to) a wide range of voluntary gross and fine motor movement such as tapping, arm raises, swaying, walking, or the use of movement to express internal states and emotions. [33-35]
**Imagery**	Mental images experienced spontaneously or in response to suggestion or some other form of sensory stimuli.
**Relaxation**	Activities or practices intended to relieve tension. These may include but are not limited to mindfulness, meditation, and breathwork.
**Other Arts Media**	Refers to arts media other than music. These may include but are not limited to storytelling, drama, visual arts, dance, or virtual reality.
**Spiritual Practice**	Activities and rituals used to deepen one's relationship or sense of connection with nature, the sacred/divine, a higher power(s), and/or the universe. Practices may include but are not limited to prayer, time in nature, meditation, and worship.
**Analysis/Discussion**	The act or process of discussing or examining something to gain clarity or insight, reach a decision, and/or exchange ideas.
**Biofeedback**	The process of training someone to acquire voluntary control of a typically involuntary physiological or brain function through closely monitoring that function.
**Human Touch**	The act or process of providing wanted, supportive, intentional forms of human touch, including but not limited to massage, comfort positioning, and holding a person.

Table References

1. Latham A. The Oxford companion to music. 2002.
2. LaRue J. Guidelines for style analysis. 2. painos. Warren: Harmonie Park Press; 1992.
3. Fabbri F. A theory of musical genres. Two applications. Popular music perspectives: Iaspm; 1982: 52-81.
4. Hyer B. Tonality. Grove Music Online. Oxford Music Online; 2012.
5. Truax B. Handbook for acoustic ecology: Cambridge Street Publishing; 1999.
6. Crombie D. New Complete Synthesizer. London, United Kingdom: Omnibus Press; 1986.
7. Redon M. What is tonality in product sound design? https://www.ansys.com/blog/what-is-tonality (accessed July 7 2025).
8. Daniel P. Roughness perception: Acoustic and psychacoustic correlates. *Journal of the Acoustical Society of America* 2008: 263-4.
9. Huang N, Elhilali M. Auditory salience using natural soundscapes. *The Journal of the Acoustical Society of America* 2017; **141**(3): 2163-76.
10. Greasley A, & Lamont, A. Music preferences. In: S. Hallam IC, & M. Thaut, ed. The Oxford Handbook of Music Psychology. 2nd ed. Oxford, United Kindgom: Oxford University Press; 2016: 263-81.
11. Mehdizadeh SK, Leslie G. The Physiology of Musical Preference: A Secondary Analysis of the Study Forrest Dataset. *Music Perception: An Interdisciplinary Journal* 2023; **40**(5): 395-409.
12. Rentfrow PJ, Goldberg LR, Levitin DJ. The structure of musical preferences: a five-factor model. *Journal of personality and social psychology* 2011; **100**(6): 1139.
13. Jagiello R, Pomper U, Yoneya M, Zhao S, Chait M. Rapid brain responses to familiar vs. unfamiliar music–an EEG and pupillometry study. *Scientific reports* 2019; **9**(1): 15570.
14. King E, Prior HM. Music and familiarity: Listening, musicology and performance: Routledge; 2016.
15. Meyer M. Experimental studies in the psychology of music. *The American Journal of Psychology* 1903; **14**(3/4): 192-214.
16. Kraus N, Chandrasekaran B. Music training for the development of auditory skills. *Nature reviews neuroscience* 2010; **11**(8): 599-605.
17. Janata P, Tomic ST, Rakowski SK. Characterisation of music-evoked autobiographical memories. *Memory* 2007; **15**(8): 845-60.
18. Warrenburg LA. Choosing the right tune: A review of music stimuli used in emotion research. *Music perception* 2020; **37**(3): 240-58.
19. Reybrouck M, Vuust P, Brattico E. Neural correlates of music listening: Does the music matter? *Brain Sciences* 2021; **11**(12): 1553.
20. Bruscia K. Defining Music Therapy. 3rd ed. Gilsum, NH: Barcelona Publishers; 2014.
21. Lehmberg LJ. Music instructional methods. Encyclopedia of the Sciences of Learning: Springer; 2012: 2404-8.
22. Siedenburg K, Bürgel M, Özgür E, Scheicht C, Töpken S. Vibrotactile enhancement of musical engagement. *Scientific Reports* 2024; **14**(1): 7764.
23. Robb SL, Story KM, Harman E, et al. Reporting Guidelines for Music-based Interventions checklist: explanation and elaboration guide. *Frontiers in Psychology* 2025; **16**: 1552659.

24. Perkins R, Mason-Bertrand A, Fancourt D, Baxter L, Williamon A. How participatory music engagement supports mental well-being: a meta-ethnography. *Qualitative health research* 2020; **30**(12): 1924-40.
25. Robb SL, Springs S, Edwards E, et al. Reporting Guidelines for Music-based Intervention: an update and validation study. *Frontiers in psychology* 2025; **16**: 1551920.
26. Loewy J. Music Psychotherapy Assessment. *Music Therapy Perspectives* 2000; **18**(1): 47-58.
27. Loewy JV, Rossetti A, Telsey A, Dassler AM. Assessing & treating trauma: Music psychotherapy for parents of neonates. *Music and Medicine* 2021; **13**(2): 138-49.
28. Raglio A, Bellandi D, Gianotti M, et al. Daily music listening to reduce work-related stress: a randomized controlled pilot trial. *Journal of Public Health* 2020; **42**(1): e81-e7.
29. Xu L, Wen X, Shi J, et al. Effects of individual factors on perceived emotion and felt emotion of music: based on machine learning methods. *Psychology of Music* 2021; **49**(5): 1069-87.
30. Lepping RJ, Hess BJ, Taylor JM, Hanson-Abromeit D, Williams KN. Inconsistent Music-Based Intervention Reporting in Dementia Studies: A Systematic Mapping Review. *J Alzheimers Dis* 2024; **100**(4): 1145-59.
31. Robb SL, Hanson-Abromeit D, May L, et al. Reporting quality of music intervention research in healthcare: A systematic review. *Complement Ther Med* 2018; **38**: 24-41.
32. Rodwin AH, Shimizu R, Travis Jr R, James KJ, Banya M, Munson MR. A systematic review of music-based interventions to improve treatment engagement and mental health outcomes for adolescents and young adults. *Child and Adolescent Social Work Journal* 2023; **40**(4): 537-66.
33. Cruz-Garza JG, Hernandez ZR, Nepaul S, Bradley KK, Contreras-Vidal JL. Neural decoding of expressive human movement from scalp electroencephalography (EEG). *Frontiers in human neuroscience* 2014; **8**: 188.
34. Virameteekul S, Bhidayasiri R. We move or are we moved? Unpicking the origins of voluntary movements to better understand semivoluntary movements. *Frontiers in neurology* 2022; **13**: 834217.
35. Gonzalez-Sanchez VE, Zelechowska A, Jensenius AR. Correspondences between music and involuntary human micromotion during standstill. *Frontiers in psychology* 2018; **9**: 355448.
